# Choosing the Right Life Partner: Ecological Drivers of Lichen Symbiosis

**DOI:** 10.3389/fmicb.2021.769304

**Published:** 2021-12-14

**Authors:** Lucie Vančurová, Jiří Malíček, Jana Steinová, Pavel Škaloud

**Affiliations:** ^1^Department of Botany, Faculty of Science, Charles University, Prague, Czechia; ^2^Institute of Botany, The Czech Academy of Sciences, Průhonice, Czechia

**Keywords:** *Asterochloris mediterranea*, lichen, Macaronesia, phycobiont sharing, specificity, *Stereocaulon canariense*, symbiosis, temperature gradient

## Abstract

Lichens are an iconic example of symbiotic systems whose ecology is shaped by the requirements of the symbionts. Previous studies suggest that fungal (mycobionts) as well as photosynthesizing (phycobionts or cyanobionts) partners have a specific range of acceptable symbionts that can be chosen according to specific environmental conditions. This study aimed to investigate the effects of climatic conditions and mycobiont identity on phycobiont distribution within the lichen genera *Stereocaulon*, *Cladonia*, and *Lepraria*. The study area comprised the Canary Islands, Madeira, Sicily, and the Aeolian Islands, spanning a wide range of climatic conditions. These islands are known for their unique and diverse fauna and flora; however, lichen phycobionts have remained unstudied in most of these areas. In total, we genetically analyzed 339 lichen samples. The phycobiont pool differed significantly from that outside the studied area. *Asterochloris mediterranea* was identified as the most abundant phycobiont. However, its distribution was limited by climatic constraints. Other species of *Asterochloris* and representatives of the genera *Chloroidium*, *Vulcanochloris*, and *Myrmecia* were also recovered as phycobionts. The selection of symbiotic partners from the local phycobiont pool was driven by mycobiont specificity (i.e., the taxonomic range of acceptable partners) and the environmental conditions, mainly temperature. Interestingly, the dominant fungal species responded differently in their selection of algal symbionts along the environmental gradients. *Cladonia rangiformis* associated with its phycobiont *A. mediterranea* in a broader range of temperatures than *Stereocaulon azoreum*, which favors other *Asterochloris* species along most of the temperature gradient. *Stereocaulon vesuvianum* associated with *Chloroidium* spp., which also differed in their temperature optima. Finally, we described *Stereocaulon canariense* as a new endemic species ecologically distinct from the other Stereocaulon species on the Canary Islands.

## Introduction

The Canary Islands, Madeira, Sicily, and the Aeolian Islands are known for their unique and diverse fauna and flora (Troia, 2012; Di Gristina et al., 2016; Giovino et al., 2016; del Arco Aguilar and Rodríguez Delgado, 2018). Macaronesia is also known for its many endemic lichens (Sérusiaux et al., 2011; Sparrius et al., 2017; Vondrák et al., 2020), and numerous lichen taxa from this area have been described (Crespo et al., 2006; Hafellner, 2008; Hernández Padrón and Pérez-Vargas, 2010; van den Boom and Ertz, 2013; van den Boom and Clerc, 2015; van den Boom and Magain, 2020; Vondrák et al., 2020). A substantial elevation gradient is typical in most of these areas; therefore, the climatic conditions are diverse despite the relatively small area of the islands. The vegetation is clearly divided into altitudinal belts. These belts are asymmetrically developed on the windward (cooler, more humid and rainier, and more climatically diverse) and leeward (warmer and drier) slopes (del Arco Aguilar and Rodríguez Delgado, 2018). All these islands, except for Sicily, are of volcanic origin.

Lichens are a classic example of symbiotic systems growing worldwide from polar to tropical regions. Due to their symbiotic nature, they can cope well with harsh conditions and newly formed bare substrates. The genera *Cladonia*, *Lepraria*, and *Stereocaulon* belong to the two closely related families Cladoniaceae (*Cladonia*) and Stereocaulaceae (*Stereocaulon* and *Lepraria*; Lücking, 2019). These mycobionts are known to preferably associate with *Asterochloris* phycobionts (Piercey-Normore and DePriest, 2001; Yahr et al., 2004; Nelsen and Gargas, 2006, 2008; Beiggi and Piercey-Normore, 2007; Bačkor et al., 2010; Škaloud and Peksa, 2010; Peksa and Škaloud, 2011). When describing the relationships between phycobionts and mycobionts, the terms specificity and selectivity are frequently used. The specificity delimits the taxonomic range of acceptable partners, whereas selectivity refers to the preference for a particular group of partners (Rambold et al., 1998; Yahr et al., 2004, 2006). Particular species of *Cladonia*, *Lepraria*, and *Stereocaulon* differ in their preferred *Asterochloris* species-level lineages (i.e., they are selective towards their phycobionts; Peksa and Škaloud, 2011; Škaloud et al., 2015; Steinová et al., 2019; Vančurová et al., 2020). A relatively high number of studies have focused on phycobionts of *Cladonia*, *Lepraria*, and *Stereocaulon*; however, new phycobiont species-level lineages continue being discovered (Moya et al., 2015; Vančurová et al., 2018; Pino-Bodas and Stenroos, 2020; Kosecka et al., 2021). In addition, other trebouxiophycean algal genera have been detected as phycobionts of *Stereocaulon* (Beck, 2002; Vančurová et al., 2015, 2018, 2020; Kosecka et al., 2021), *Cladonia* (Ahmadjian and Jacobs, 1981; Korchikov et al., 2018; Osyczka et al., 2020), and *Lepraria* (Engelen et al., 2010). The ability to associate with an alternative photobiont (i.e., lower selectivity) can be caused by a stressful environment, as previously shown (Romeike et al., 2002; Engelen et al., 2010; Osyczka et al., 2020).

Since lichen phycobionts in this region have remained almost unresearched (past studies involved only a limited number of samples; Muggia et al., 2014; Leavitt et al., 2015; Moya et al., 2015, 2018; Boluda et al., 2019; Molins et al., 2020), our aim was to supplement the missing information on photobiont diversity in this study area and explore ecological factors shaping symbiotic relationships. Moreover, several studies have documented the phycobionts that are shared by diverse lichen species/genera (Bačkor et al., 2010; Vargas Castillo and Beck, 2012; Peksa et al., in preparation), whereas other studies failed to search for the source of lichen phycobionts (Vančurová et al., 2020). Therefore, we also examined the sharing of phycobionts within the lichen community. This is closely related to the level of specificity and selectivity of the symbiotic partners. Hence, the main questions we addressed were what are (1) the patterns of phycobiont diversity within the study area, (2) the shared pool of phycobionts, and (3) the effect of the climatic conditions on the symbiotic relationships.

## Materials and Methods

### Taxon Sampling

We analyzed a total of 339 specimens (Supplementary Table 1) belonging to three closely related fungal genera *Cladonia* (*n*=179), *Lepraria* (*n*=23), and *Stereocaulon* (*n*=137) collected between years 2011 and 2020 on the Canary Islands (El Hierro, La Palma, La Gomera, Tenerife, Gran Canaria, Lanzarote), Madeira, Sicily, and the Aeolian Islands (Vulcano, Salina, Stromboli; Supplementary Figure 1). The sampling sites represented diverse habitats from malpaíses (i.e., barren landscapes) to a laurel forest, various types of substrates, and were located at a range of altitudes 75–2,360m. We identified the mycobiont morphospecies using standard morphological methods, taxonomical keys (e.g., Lamb, 1978; Baruffo et al., 2006; Burgaz and Ahti, 2009) and thin-layer chromatography (TLC) on Merck silica gel 60 F254 pre-coated glass plates in solvent systems A, B, and C, according to Orange et al. (2001). For the purpose of describing *Stereocaulon* species, measurements and observations were done in water, except for ascospores and paraphyses observed in KOH. Lichen specimens were deposited in the Herbarium of the Institute of Botany, the Czech Academy of Sciences (PRA), and the private herbarium of J. Malíček.

### DNA Extraction, Amplification, and Sequencing

We extracted DNA directly from lichen thalli (total lichen DNA) following the standard CTAB protocol for lichens (Cubero et al., 1999). Firstly, we examined thalli under a dissecting microscope and washed them with running water (except for *Lepraria* thalli) before DNA extraction to prevent contamination by soredia from other lichens. We amplified the algal and fungal nuclear internal transcribed spacer (ITS, ITS1-5.8S-ITS2 rDNA) and the algal actin type I gene (including one complete exon and two introns located at codon positions 206 and 248; Weber and Kabsch, 1994) using primers listed in Table 1. The PCR conditions were as described in Vančurová et al. (2018). Every PCR run included negative controls, without a DNA template. The PCR products were purified using by NucleoMag® NGS Clean-up and Size Select kit (Macherey-Nagel, Duren, Germany) and sequenced using the same primers at Macrogen in Amsterdam, Netherlands. The 507 newly obtained sequences of the ITS rDNA and actin type I regions are available in GenBank under accession numbers OL622077–OL622095 and OL625120–OL625607 (Supplementary Table 1).

**Table 1:** Primers used in this study.

Name	Sequence	Function	References
nr-SSU-1780-5'	5'-CTG CGG AAG GAT CAT TGA TTC-3'	Algal ITS region, algal-specific	Piercey-Normore and DePriest, 2001
ITS1-F-5'	5'-CTT GGT CAT TTA GAG GAA GTA A-3'	Fungal ITS region, fungal-specific	Gardes and Bruns, 1993
ITS4-3'	5'-TCC TCC GCT TAT TGA TAT GC-3'	Algal and fungal ITS region, universal	White et al., 1990
a-nu-act1-0645-5'	5'-GAC AGA GCG TGG KTA CAG-3'	Actin type I locus, algal specific	Nelsen and Gargas, 2006
a-nu-act1-0818-3'	5'-TGA ACA GCA CCT CAG GGC A-3'	Actin type I locus, algal specific	Nelsen and Gargas, 2006
ActinF2 Astero-5'	5'-AGC GCG GGT ACA GCT TCA C-3'	Actin type I locus, algal specific	Škaloud and Peksa, 2010
ActinR2 Astero-3'	5'-CAG CAC TTC AGG GCA GCG GAA-3'	Actin type I locus, algal specific	Škaloud and Peksa, 2010
ActinF Astero-5'	5'-GGG TAC AGC TTC AC-3'	Actin type I locus, algal specific	Vančurová et al., 2018
ActinR Astero-3'	5'-TGA ACA GCA CTT CAG GGC A-3'	Actin type I locus, algal specific	Vančurová et al., 2018
ActinF3 Astero-5'	5'-AGC TTC ACC ACC ACT GCA G-3'	Actin type I locus, algal specific	Vančurová et al., 2018
ActinR3 Astero-3'	5'-AGC GGA AKC GCT CGC TGC C-3'	Actin type I locus, algal specific	Vančurová et al., 2018

### Sequence Alignment and DNA Analyses

We prepared individual sequence alignments for *Stereocaulon*, *Cladonia*, *Lepraria*, *Chloroidium*, *Vulcanochloris* (ITS rDNA), and *Asterochloris* (ITS rDNA and actin type I) datasets using MAFFT v.7 software (Katoh and Standley, 2013). The sequences obtained for *Asterochloris* were analyzed as a single locus dataset for the ITS rDNA (data not shown) and as a concatenated dataset of ITS rDNA and actin type I loci. In the case of *Cladonia*, we used Gblocks to remove introns from alignment and to eliminate poorly aligned positions (Castresana, 2000).

Supplementary Table 2 summarizes a total number of sequences included in particular alignments, newly obtained sequences, previously published sequences originated from the study area, reference sequences retrieved from GenBank, and unique sequences after deleting identical ones. All DNA alignments are freely available on Mendeley Data: http://dx.doi.org/10.17632/428v52svtp.1.

Phylogenetic trees were inferred with Bayesian Inference (BI) using MrBayes v.3.2.7a (Ronquist and Huelsenbeck, 2003; Ronquist et al., 2012), maximum likelihood (ML) analysis using GARLI v.2.0 (Zwickl, 2006), and maximum parsimony (MP) analysis using PAUP v.4.0b10 (Swofford, 2003). BI and ML analyses were carried out on a partitioned dataset to differentiate among ITS1, 5.8 S and ITS2 rDNA, actin intron 206, actin intron 248, and actin exon regions. Substitution models (Supplementary Table 3) were selected using the Bayesian information criterion (BIC) as implemented in JModelTest2 (Guindon and Gascuel, 2003; Darriba et al., 2012). Two parallel MCMC runs, with four chains, were carried out for 10 and 5 million generations for *Asterochloris* and other datasets, respectively. Trees and parameters were sampled every 100 generations. Convergence of the two cold chains was assessed during the run by calculating the average SD of split frequencies (SDSF). The SDSF value between simultaneous runs was < 0.01 in all cases. Finally, the burn-in values were determined using the “sump” command. ML analysis was carried out using default settings, five search replicates, and the automatic termination set at 5 million generations. The MP analysis was performed using heuristic searches with 1,000 random sequence addition replicates and random addition of sequences (the number was limited to 10^4^ per replicate). ML and MP bootstrap support values were obtained from 100 to 1,000 bootstrap replicates, respectively. Only one search replicate was applied for ML bootstrapping.

We maintained the nomenclature and species boundaries delimited in recent studies (Škaloud et al., 2015; Vančurová et al., 2018, 2020; Kosecka et al., 2021) for *Asterochloris* species and the species boundaries delimited by Vančurová et al. (2018) and the nomenclature used therein (OTU1–OTU57) for *Stereocaulon* species-level lineages.

### Interaction Networks

We depicted the interactions between phycobiont species-level lineages in the genus *Asterochloris* and mycobiont genera and between phycobiont species-level lineages and mycobiont species-level lineages in the genus *Stereocaulon* as interaction networks produced using bipartite package (Dormann et al., 2008) in R.

### Niche Hypervolumes

The climatic niche of the most abundant species-level lineages of phycobionts and *Stereocaulon* mycobionts were represented using the Hutchinsonian niche concept that describes a species niche as an n-dimensional hypervolume, where the dimensions are environmental variables (Hutchinson, 1957). In the present study, these environmental dimensions were defined based on 19 Bioclim variables (Karger et al., 2017). We constructed the climatic hypervolumes by multivariate kernel density estimation (Blonder et al., 2014). First, we performed the PCA analysis of 19 Bioclim variables to reduce the total number of predictors. The first two PCA axes (explaining 82% of the total variance) were then selected to calculate hypervolumes for each species-level lineage and genus. The boundaries of the kernel density estimates were delineated by the probability threshold, using the 0.85 quantile value. To project the niche spaces of particular lineages, hypervolume contours were plotted based on 5,000 random background points, using the alphahull contour type and alpha smoothing value 0.55. The analyses were performed in R, using the hypervolume (Blonder et al., 2014) and alphahull (Pateiro-Lopez and Rodriguez-Casal, 2016) packages.

### Statistical Comparisons of Contrasting Phycobiont Groups

To assess the influence of climatic variables on phycobiont distribution, the contrasting phycobiont groups were compared for each Bioclim variable and altitude using Welch’s two-sample *t*-test. We compared: (1) *Asterochloris mediterranea* (as the most abundant phycobiont in the study area) with other *Asterochloris* spp. in association with all mycobionts, (2) *A. mediterranea* with other *Asterochloris* spp. in association with *Cladonia rangiformis* (as the most abundant *Cladonia* species in the study area) mycobiont, (3) *A. mediterranea* with other *Asterochloris* spp. in association with *Stereocaulon azoreum* mycobiont (as the most abundant *Stereocaulon* species in the study area associated with *Asterochloris* spp.), and (4) *Chloroidium ellipsoideum* with *C. lichenum* A (sensu Darienko et al., 2018) in association with *Stereocaulon vesuvianum* (OTU11) mycobiont (as the most abundant *Stereocaulon* species in the study area). We tested phycobionts in pairs with their mycobionts to inspect ecological requirements of the symbiotic system (unlike to separate analyses of symbiotic partners in previous studies, e.g., Vančurová et al., 2018). We could not compare those groups of phycobionts associated with *Lepraria* spp. mycobionts due to the low number of samples. We performed the analyses in R using function t.test.

## Results

### Diversity of Mycobionts

**Figure 1:**
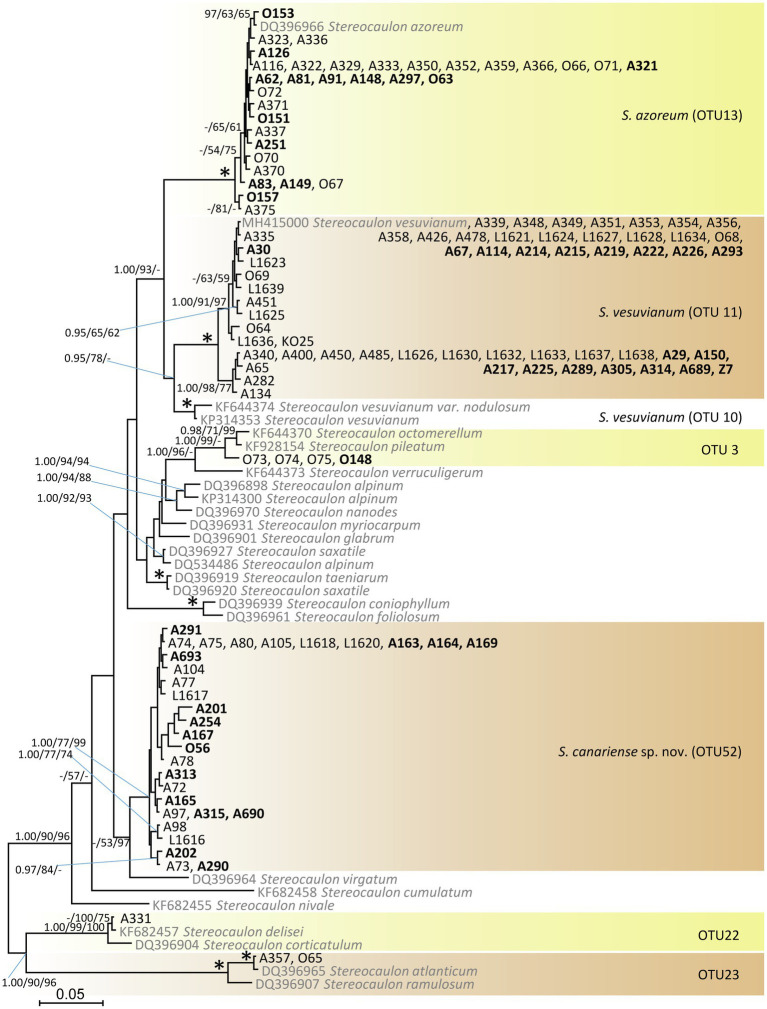
Phylogenetic hypothesis (unrooted tree) of *Stereocaulon* resulting from Bayesian analysis of ITS rDNA. Values at the nodes indicate the statistical supports of Bayesian posterior probability (left), maximum-likelihood bootstrap (middle) and maximum parsimony bootstrap (right). Fully supported branches (1.0/100/100) are marked with an asterisk. Scale bar shows the estimated number of substitutions per site. Newly obtained sequences are marked in bold. We selected the reference sequences from GenBank (in grey) taking care to cover eight main *Stereocaulon* clades (Högnabba, 2006).

We recovered *Stereocaulon* mycobionts from the study area in six lineages (Figure 1): OTU13 (identified morphologically as *S. azoreum* and clustered with *S. azoreum* sequences), OTU11 (identified morphologically as *S. vesuvianum*), OTU3 (identified morphologically as *S. pileatum* and clustered with *S. pileatum* and *S. octomerellum*), OTU52 (described here as a new species *Stereocaulon canariense*; sister to *S. virgatum* sequence DQ396964), OTU22 (clustered with *S. delisei* and *S. corticatulum*), and OTU23 (clustered with *S. atlanticum* and *S. ramulosum*). The most frequent species in the study area was *S. vesuvianum* (47% of the *Stereocaulon* samples).

A Bayesian analysis of the ITS rDNA of *Cladonia* mycobionts resulted in 22 supported *Cladonia* lineages (Supplementary Figure 2). The most recovered *Cladonia* species was *C. rangiformis* (30% of the *Cladonia* samples), followed by *C. humilis* (20% of the *Cladonia* samples). In most cases, phylogenetically separated lineages corresponded to morphologically delimited species; however, the lineage “*Cladonia* sp. 1” contained several morphologically different entities. It has been previously reported that ITS rDNA may fail to distinguish *Cladonia* species in some cases (Kelly et al., 2011; Kanz et al., 2015); however, it has been suggested as a *Cladonia* barcode marker at the same time (Pino-Bodas et al., 2013).

The phylogenetic hypothesis resulting from the Bayesian analysis of the ITS rDNA sequences of *Lepraria* (Supplementary Figure 3) was congruent with that of previous studies (Crespo et al., 2006; Tretiach et al., 2009). We recovered *Lepraria* mycobionts from the study area in five lineages. The most abundant was *L. santosii* (55% of *Lepraria* samples), originally described from Tenerife (Crespo et al., 2006).

### Diversity of Phycobionts

**Figure 2:**
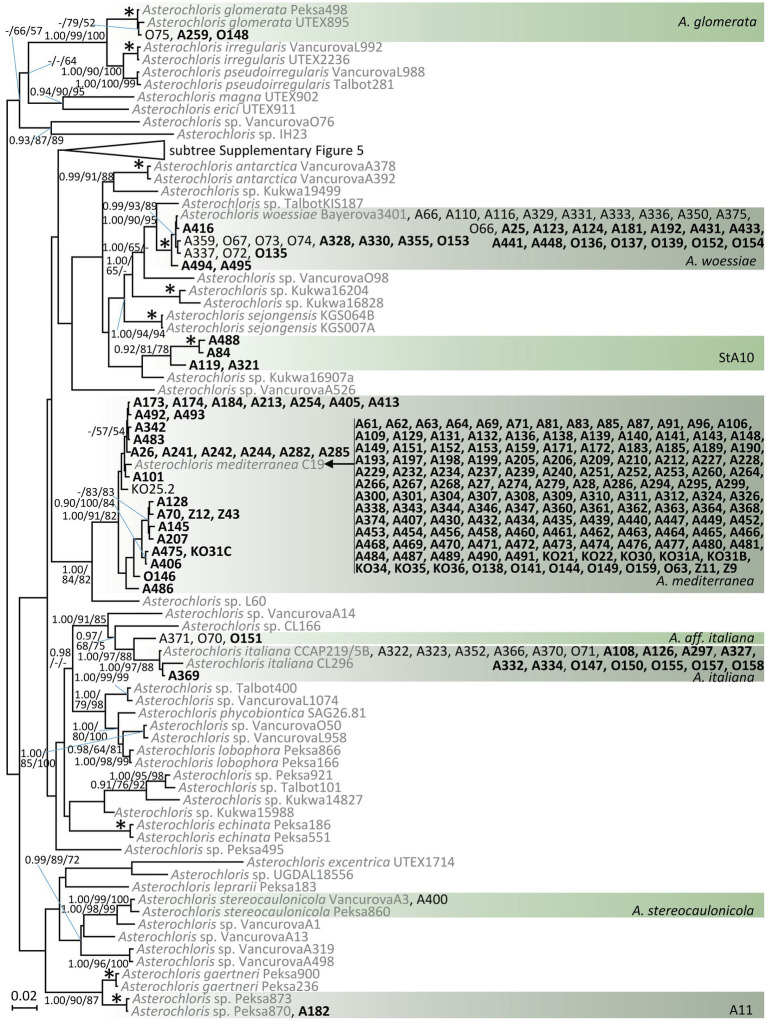
Phylogenetic hypothesis (unrooted tree) of *Asterochloris* resulting from Bayesian analysis of ITS rDNA. Values at the nodes indicate the statistical supports of Bayesian posterior probability (left), maximum-likelihood bootstrap (middle) and maximum parsimony bootstrap (right). Scale bar shows the estimated number of substitutions per site. Newly obtained sequences are marked in bold. We selected the reference sequences from GenBank (in grey; accession numbers are listed in Supplementary Table 4) taking care to include all known *Asterochloris* species as well as other previously published *Asterochloris* species-level lineages. The collapsed clade is displayed in Supplementary Figure 4.

A phylogram resulting from the Bayesian analysis of ITS rDNA and actin type I sequences of *Asterochloris* is shown in Figure 2. We recorded phycobionts from the study area in eight species-level lineages. The overwhelming majority of samples belonged to *A. mediterranea* (72% of *Asterochloris* samples). *A. woessiae* and *A. italiana* were also relatively common (16 and 7%, respectively). Other lineages were rather rare. The lineage StA10 is a new, highly resolved lineage in *Asterochloris*.

**Figure 3:**
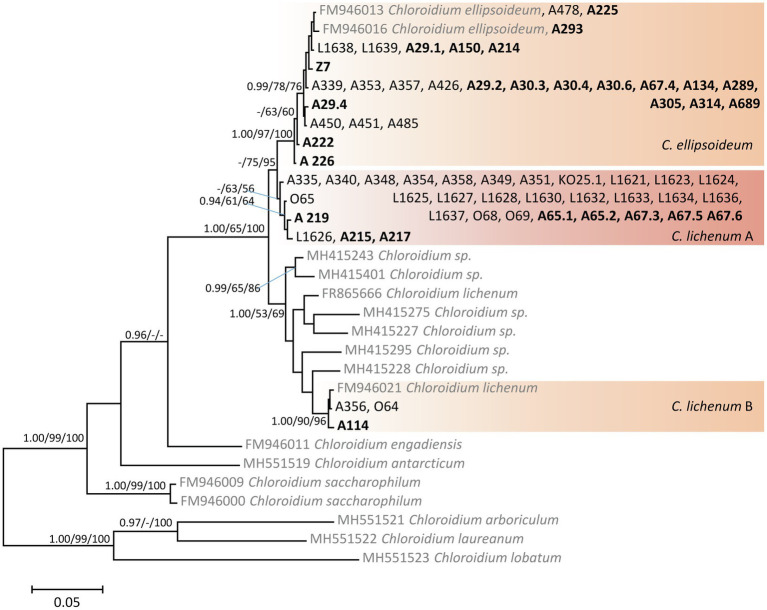
Phylogenetic hypothesis (unrooted tree) of *Chloroidium* resulting from Bayesian analysis of ITS rDNA. Values at the nodes indicate the statistical supports of Bayesian posterior probability (left), maximum-likelihood bootstrap (middle) and maximum parsimony bootstrap (right). Scale bar shows the estimated number of substitutions per site. Newly obtained sequences are marked in bold, reference sequences from GenBank are in grey.

The phylogenetic hypothesis resulting from the Bayesian analysis of the ITS rDNA sequences of *Chloroidium* (Figure 3) was congruent with that of previous studies (Darienko et al., 2018; Vančurová et al., 2018). Since *C. lichenum* became a paraphyletic species (divided into three clades: A, B, and C) after the last taxonomic revision (Darienko et al., 2018), we considered the two monophyletic clades *C. lichenum* A and *C. lichenum* B as species level-lineages (following species delimitation analyses; Vančurová et al., 2018). We recovered *Chloroidium* phycobionts from the study area in three species-level lineages: *C. ellipsoideum* (45% of *Chloroidium* samples), *C. lichenum* A (50% of *Chloroidium* samples), and *C. lichenum* B.

**Figure 4:**
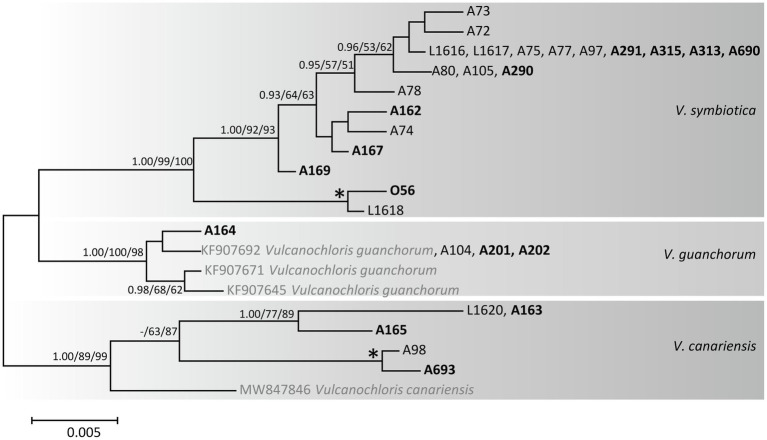
Phylogenetic hypothesis (unrooted tree) of *Vulcanochloris* resulting from Bayesian analysis of ITS rDNA. Values at the nodes indicate the statistical supports of Bayesian posterior probability (left), maximum-likelihood bootstrap (middle) and maximum parsimony bootstrap (right). Fully supported branches (1.0/100/100) are marked with an asterisk. Scale bar shows the estimated number of substitutions per site. Newly obtained sequences are marked in bold, reference sequences from GenBank are in grey.

Furthermore, we recovered *Vulcanochloris* phycobionts from the study area in three lineages corresponding to three species (Figure 4): *V. symbiotica* (70% of *Vulcanochloris* samples), *V. guanchorum*, and *V. canariensis*.

Finally, we confirmed the identity of sample A194 by a BLAST search against the GenBank database. The most similar hit (95% sequence identity) was *Myrmecia israliensis* KY981668. Therefore, we labelled this sample as *Myrmecia* sp.

### Distribution of Phycobionts

**Figure 5:**
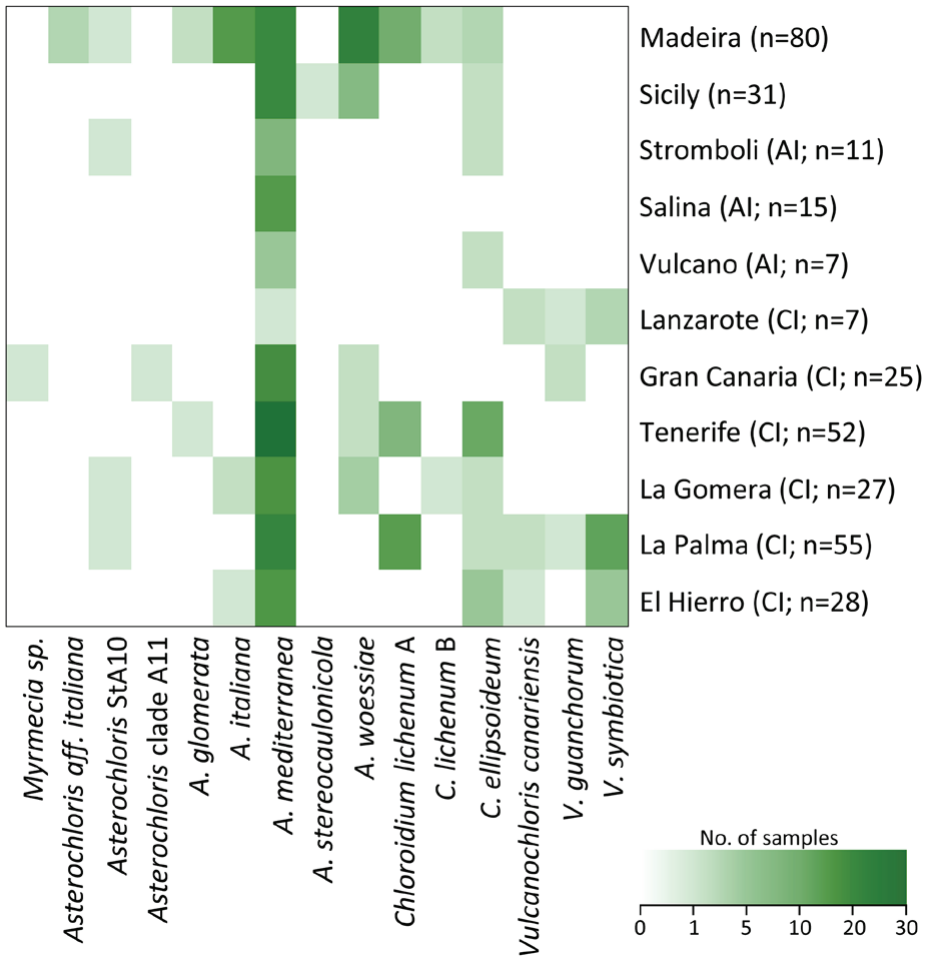
Distribution of phycobiont species-level lineages on particular islands within the study area. Number of samples is indicated in brackets. AI, Aeolian Islands and CI, Canary Islands.

In total, we found 15 species-level lineages within four genera of phycobionts, from one to nine on each island (Figure 5). Madeira was identified as the most phycobiont species-rich island in our study area. The number of species-level lineages was highest (4.1) also after rarefaction to seven samples, which was the smallest number of samples per island in the dataset.

**Figure 6:**
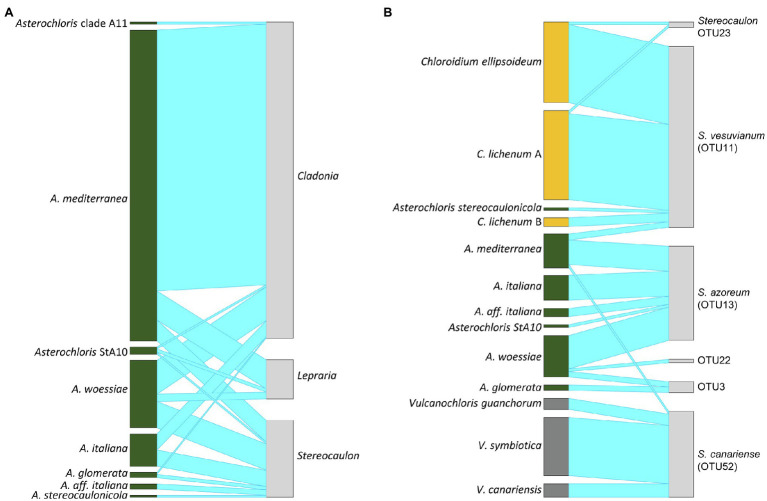
Interaction network structures: **(A)** between phycobiont species-level lineages in the genus *Asterochloris* and mycobiont genera, **(B)** between phycobiont species-level lineages and mycobiont species-level lineages in the genus *Stereocaulon*. The width of the links is proportional to the number of specimens forming the association.

*Asterochloris mediterranea* represented the most abundant phycobiont in our dataset, was present on all studied islands and formed associations with all three mycobiont genera (Figures 5, 6A). However, its prevalence in the thalli of different genera of mycobionts varied. *Asterochloris mediterranea* was present in 83, 73, and 28% of the thalli involving *Asterochloris* phycobionts in association with *Cladonia*, *Lepraria*, and *Stereocaulon*, respectively (Figure 6B). *Asterochloris woessiae* and *Asterochloris* StA10 were also shared by all three mycobiont genera and present on multiple islands. On the other hand, *A. italiana* and *A. glomerata* associated solely with representatives of *Stereocaulon* and *Cladonia*.

*Chloroidium ellipsoideum* associated with *S. vesuvianum* and *Stereocaulon* OTU23 (Figures 5, 6B), was the second most geographically widespread phycobiont, and was present on eight islands (absent on Gran Canaria, Lanzarote, and Isola Salina).

The phycobionts *Myrmecia* sp. (associated with a *Cladonia* mycobiont), *Asterochloris* clade A11 (with *Cladonia*), and *A. stereocaulonicola* (with *Stereocaulon*) were recorded only once in our dataset. The phycobiont *A. aff. italiana* was found in three samples but only on a single island (Madeira) and was associated with a single mycobiont (*Stereocaulon azoreum*).

*Vulcanochloris* phycobionts were restricted to the Canary Islands, namely La Palma, Lanzarote, El Hierro, and Gran Canaria. On La Palma and Lanzarote, all three *Vulcanochloris* species were present. Interestingly, *Vulcanochloris* phycobionts were found exclusively in association with *S. canariense* (Figure 6B).

### Climatic Niches

**Figure 7:**
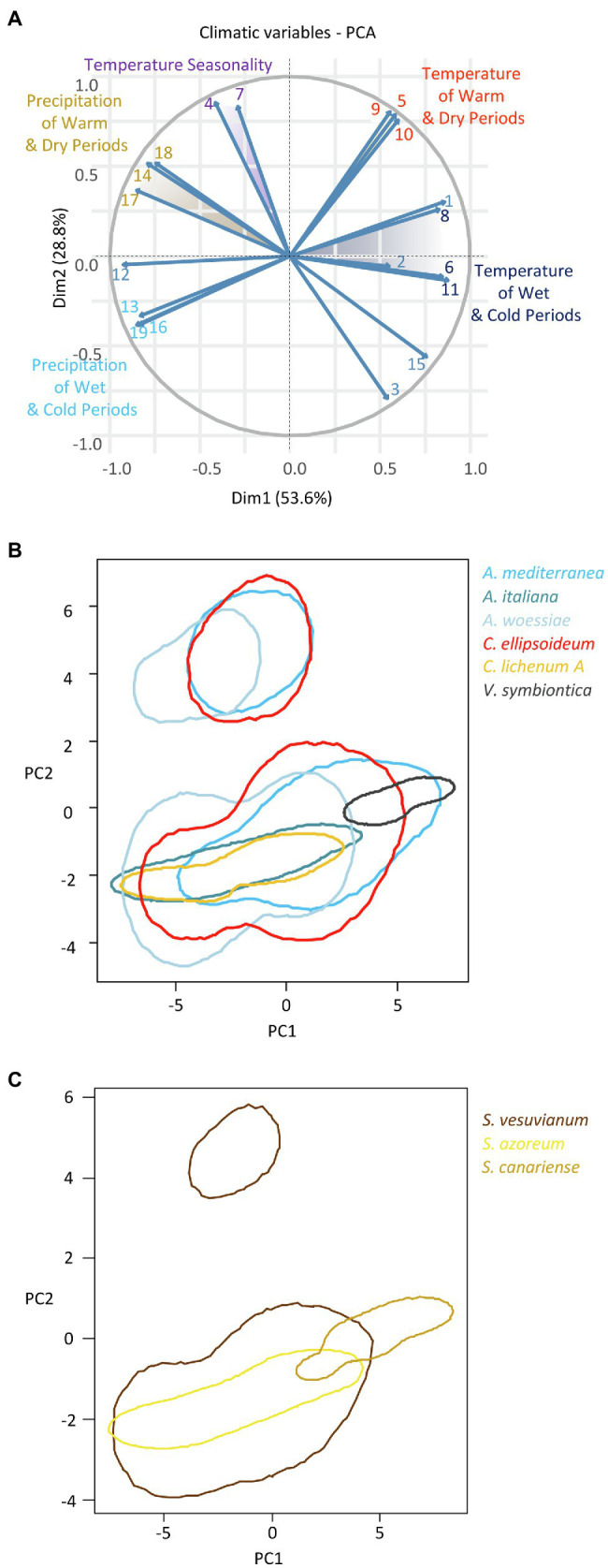
**(A)** Principal coordinate analysis of 19 Bioclim variables: 1=annual mean temperature, 2=mean diurnal range, 3=isothermality, 4=temperature seasonality, 5=max temperature of warmest month, 6=min temperature of coldest month, 7=temperature annual range, 8=mean temperature of wettest quarter, 9=mean temperature of driest quarter, 10=mean temperature of warmest quarter, 11=mean temperature of coldest quarter, 12=annual precipitation, 13=precipitation of wettest month, 14=precipitation of driest month, 15=precipitation seasonality, 16=precipitation of wettest quarter, 17=precipitation of driest quarter, 18=precipitation of warmest quarter, 19=precipitation of coldest quarter. Climatic niche hypervolumes for **(B)** six most abundant phycobiont species-level lineages and **(C)** three most abundant *Stereocaulon* mycobiont species-level lineages.

We constructed two-dimensional (PC1-PC2, explaining 82.4% of the variation in climatic variables) hypervolumes for the six most abundant algal species-level lineages and the three most abundant *Stereocaulon* mycobiont species-level lineages. Since the climatic characteristics differed considerably between Macaronesia and the Mediterranean region, the hypervolumes split into two isolated parts. Within each of them, there was a gradient of climatic niches along PC1, representing mostly the gradients of temperature and precipitation (Figure 7).

Among the algal species-level lineages, *Vulcanochloris symbiotica* tolerated the driest and warmest climate. On the other hand, *Asterochloris woessiae*, *A. italiana*, and *Chloroidium lichenum* A were distributed predominantly in the more humid and relatively colder areas. *Asterochloris mediterranea* and *C. ellipsoideum* were widely distributed in warm and dry areas and their climatic niches partly overlapped with that of both sides of the continuum. Climatic niches of *A. mediterranea*, *C. ellipsoideum*, and *A. woessiae* involved the whole range of temperature and precipitation seasonality (corresponding roughly with PC2). Remarkably, the niches of *A. mediterranea* and *C. ellipsoideum* overlapped to a large extent. Similarly, the niches of *A. italiana* and *C. lichenum* A were equivalent.

The climatic hypervolume of *S. vesuvianum* was the widest among *Stereocaulon* species. It included the whole range of temperature seasonality in Macaronesia as well as that of the Mediterranean region. The warmest and driest areas with high precipitation seasonality were dominated by *S. canariense*. The climatic niche of *S. azoreum* overlapped with part of that of *S. vesuvianum*. These two species likely differed in microclimatic factors (not included in this study).

**Figure 8:**
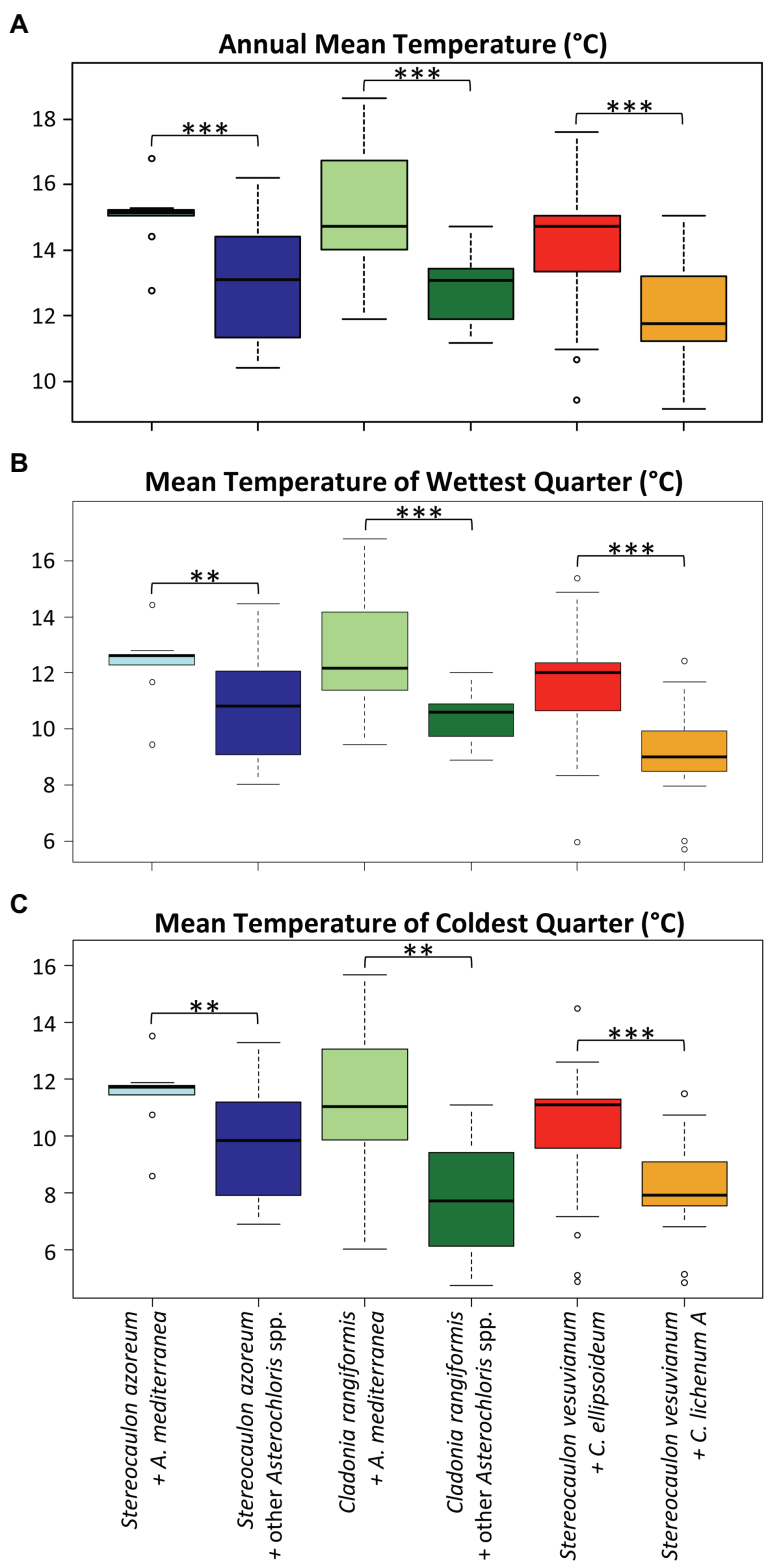
Differences in the distribution of selected phycobiont-mycobiont pairs along the gradients of **(A)** annual mean temperature, **(B)** mean temperature of wettest quarter, and **(C)** mean temperature of coldest quarter. Significance level: two stars (**): *p*<0.01, three stars (***): *p*<0.001.

Finally, we focused on the niche of the most abundant phycobiont in the study area, *A. mediterranea*. We inspected the differences between the climatic requirements of this species and those of other *Asterochloris* species associated with either two dominant mycobiont species (*Cladonia rangiformis* and *Stereocaulon azoreum*) or all mycobionts using Welch’s *t*-tests. Similarly, we tested the differences between *Chloroidium ellipsoideum* and *C. lichenum* A. Table 2 summarizes the results of the tests performed for various combinations of phycobionts and mycobionts. The means of three variables showed significant differences (*α*=0.01) across all combinations: BIO1 – annual mean temperature, BIO8 – mean temperature of the wettest quarter, and BIO11 – mean temperature of the coldest quarter (Figure 8). In contrast, we did not find any significant difference between the groups in BIO3 [Isothermality (BIO2/BIO7)] and BIO4 (Temperature Seasonality) climatic variables.

**Table 2:** Results of Welch’s *t*-tests (*p*) performed for contrasting pairs of phycobionts in combination with their mycobionts.

Climatic variable	*Asterochloris mediterranea* with other *Asterochloris* spp. in association with all mycobionts	*Asterochloris mediterranea* with other *Asterochloris* spp. in association with *Cladonia rangiformis*	*Asterochloris mediterranea* with other *Asterochloris* spp. in association with *Stereocaulon azoreum*	*Chloroidium ellipsoideum* with *C. lichenum* A in association with *Stereocaulon vesuvianum*
BIO1	3.9e^−16^	0.000108	0.000252	3.861 e^−06^
BIO2	1.221 e^−07^	0.8077	6.786 e^−07^	0.4361
BIO3	0.4156	0.1005	0.7618	0.2455
BIO4	0.0481	0.1083	0.0193	0.108
BIO5	<2.2 e^−16^	0.3672	1.888 e^−09^	5.156 e^−06^
BIO6	6.366 e^−07^	0.0005975	0.01907	0.0003686
BIO7	0.001919	0.1031	0.0001044	0.06981
BIO8	1.728 e^−14^	1.019 e^−05^	0.009172	5.103 e^−06^
BIO9	<2.2 e^−16^	0.3203	7.36 e^−07^	4.267 e^−06^
BIO10	<2.2 e^−16^	0.1695	2.584 e^−06^	3.523 e^−06^
BIO11	1.231 e^−07^	0.001109	0.003458	0.0003848
BIO12	5.331 e^−13^	0.1466	3.869 e^−10^	0.02286
BIO13	7.446 e^−13^	0.3267	1.421 e^−06^	7.618 e^−05^
BIO14	9.298 e^−06^	0.07341	4.231 e^−10^	0.9307
BIO15	7.683 e^−05^	0.09019	5.233 e^−12^	0.7347
BIO16	1.527 e^−13^	0.3982	1.63 e^−07^	6.214 e^−05^
BIO17	1.337 e^−08^	0.065	9.763 e^−11^	0.617
BIO18	9.482 e^−05^	0.1287	4.408 e^−11^	0.9033
BIO19	2.164 e^−14^	0.379	5.673 e^−07^	3.624 e^−05^
Altitude	2.977 e^−08^	0.05307	0.09813	0.003085

Bioclim variables: 1=annual mean temperature, 2=mean diurnal range, 3=isothermality, 4=temperature seasonality, 5=max temperature of warmest month, 6=min temperature of coldest month, 7=temperature annual range, 8=mean temperature of wettest quarter, 9=mean temperature of driest quarter, 10=mean temperature of warmest quarter, 11=mean temperature of coldest quarter, 12=annual precipitation, 13=precipitation of wettest month, 14=precipitation of driest month, 15=precipitation seasonality, 16=precipitation of wettest quarter, 17=precipitation of driest quarter, 18=precipitation of warmest quarter, 19=precipitation of coldest quarter.

Interestingly, along the gradient of annual mean temperature, *A. mediterranea* was replaced by other *Asterochloris* species at different temperature levels depending on whether it was associated with *Stereocaulon azoreum* or *C. rangiformis* (the later in about 1°C colder temperature). Furthermore, *C. ellipsoideum* was replaced by *C. lichenum* A at an annual mean temperature of approximately 13°C (Figure 8).

### Taxonomy

*Stereocaulon canariense* Malíček & Vančurová sp. nov.

Mycobank: MB841859.

#### Type

Spain, Canary Islands. El Hierro, Parque rural de Frontera, Ermita de los Reyes, N-facing lava field, 27°43′48.9″N, 18°07′13.6″W, alt. 710m, on lava rock, leg. L. Vančurová & J. Malíček 16. 5. 2013 (holotype – PRA-00020984; isolate A313). ITS GenBank number OL625487.

#### Diagnosis

Almost identical with *S. vesuvianum* s. str., but calcium oxalate crystals absent from apothecia and both species differ in molecular characters of nuclear ITS region.

#### Etymology

Distribution of the new species is restricted to the Canary Islands.

#### Description

**Figure 9:**
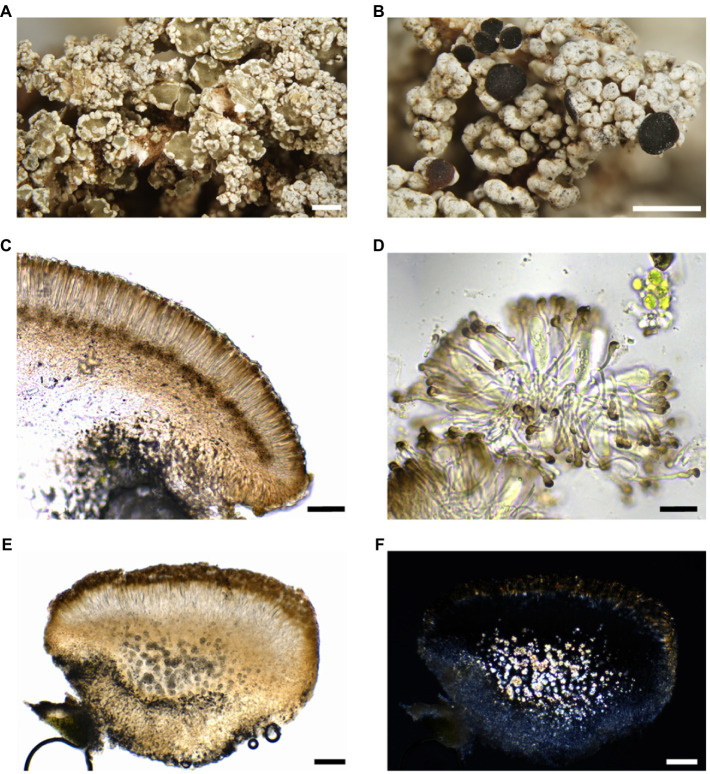
Habitus of *Stereocaulon canariense* **(A–D)** and *Stereocaulon vesuvianum* s. str. **(E,F)**. **(A)** Typical peltate phyllocladia, holotype; **(B)** fertile podetia, holotype; **(C)** section of an apothecium in water; **(D)** paraphyses with brown apical cap in KOH; **(E)** section of an apothecium in water; **(F)** section of an apothecium in polarized light. Scales: 1mm **(A,B)**, 50μm **(C–F)**, 20μm **(D)**.

Podetia up to 3.5cm, (richly) branched, smooth and without tomentum or rarely with rudimental local tomentum on the underside. Cephalodia not observed, but small bunches of *Stigonema* on podetia observed in three collections. In exposed habitats, podetia are usually low and forming more or less compact cushions covered by phyllocladia or even only basal thallus composed of phyllocladia present. Phyllocladia densely cover podetia or form basal thallus in young stages, developing from small white to pale grey granules/squamules without the dark center to typical peltate shape with a distinct white rim and olive center, resembling lecanorine apothecia, up to 1.5mm in diam (Figure 9A). Margin of phyllocladia elevated, flexuose to rarely crenulate, thick. Hyphal tissue in the olive center of phyllocladia 50–100 (−130) μm thick in section, colorless to yellowish/brownish in upper part due to crystals of atranorin (soluble in K), which are very abundant in this layer and upper part of phyllocladia margin, photobiont cells 7–13μm in diam., medullary hyphae branched, 8–15μm thick, walls 3–6μm thick.

Apothecia common in well-developed thalli, lecideine, brown to black, 0.4–0.6mm in diam., sessile to constricted at base, discs firstly plane, soon convex. Outer exciple pale brown (rarely) to brown, of thick hyphae (c. 10μm), with abundant fine crystals of atranorin (POL+, soluble in K); hypothecium very pale brown to brown, K+ yellow-brown to dark brown, without crystals; epihymenium brown, POL+ but without any crystalline objects (POL− in K); hymenium 50–75 (−100) μm. Paraphyses 1–2μm thick, sparsely branched, swollen at tips up to 4 (−5) μm, apices often with a dark brown cap. Asci 8-spored, clavate, *ca.* 35–40×8–12 (−14) μm. Ascospores 1–3-septate, (18–) 23–35×2.5–3.5μm, colorless, straight or slightly curved, with rounded apices.

Pycnidia infrequent, in phyllocladia near podetia apices, black, variable in shape, usually 100–200μm in diam. (in section), wall brown, paraplectenchymatic, conidia straight or one apex slightly curved, 5–7×1μm.

#### Chemistry

Atranorin (major), stictic acid complex (major), including norstictic acid (minor) detected by TLC (*n*=24). Spot reactions: K+ yellow, Pd+ slowly orange, C−, KC−, UV+ dull (yellow-)orange.

#### Distribution and Ecology

The new species is so far known only from the Canary Islands (Gran Canaria, El Hierro, La Palma, Lanzarote, Tenerife). Most of records come from La Palma, El Hierro and Lanzarote. It is common and widely distributed here in lower and middle elevations from 100ma.s.l. (Gran Canaria) to 960ma.s.l. (Tenerife). It occurs on lava rocks, where it is among the first colonists. In some areas, it may be a dominant in saxicolous communities. Rarely, it may grow on soil crusts in volcanic rocks or weathered lava ground.

*Caloplaca* spp. and *Candelariella vitellina* were the most commonly recorded, co-occurring species. *Acarospora* spp., *Buellia* spp., *Cladonia foliacea*, *Cetraria aculeata*, *Lecanora campestris*, and *Ramalina* spp. were other associated taxa.

#### Phylogeny

*Stereocaulon canariense* is strongly supported as a distinct clade in the ITS phylogeny; its closest relative is *S. virgatum* (Figure 1). The most similar species, *S. vesuvianum* s. str. (OTU11) and the boreal-montane to arctic-alpine *S. vesuvianum* var. *nodulosum* are (OTU10) not closely related.

#### Notes

*Stereocaulon canariense* is quite a variable species. Thalli on very dry and exposed sites are often formed by low compact cushions of podetia or even by phyllocladia alone. One sample (L1617) was composed mostly of phyllocladia, forming the basal thallus, with abundant apothecia and rare podetia up to 3mm high. In two samples (A315 and A169), local “pseudosoralia” were observed. They contained white blastidia like structures of c. 0.1mm in diam. with compact hyphae on their surface.

*Stereocaulon vesuvianum* sensu lato is a very polymorphic and cosmopolitan morphospecies well characterized by its peltate phyllocladia (grey to olive in centers) and the presence of stictic acid (Lamb, 1977). However, our molecular studies revealed three distinct lineages in the complex. Here we described only *S. canariense*, but the other taxa should be re-classified in the future. *Stereocaulon vesuvianum* sensu stricto is morphologically identical, but contains calcium oxalate crystals in lower hypothecium. We observed two additional characters, which, however, partially overlap in both species. Epihymenium in *S. vesuvianum* is usually reddish-brown (brown in *S. canariense*) and phyllocladia form generally smaller olive centers, which are often poorly developed or only punctiform comparing to usually large olive centers in *S. canariense*, and its phyllocladia strongly resembling lecanorine apothecia. Both species share similar ecology, but on the Canary Islands, they have not been collected at the same site. Most of localities of *S. vesuvianum* have been recorded above 1,000ma.s.l. (mean=1,426m, *n*=32), whereas *S. canariense* occurs below this elevation (mean=476m, *n*=25). *Stereocaulon vesuvianum* is not restricted to young stages of lava fields, but grows on various volcanic rocks also in late succession stages, e.g., well-lit forests with rocky substrata.

The most widely distributed lineage of “*S. vesuvianum*” (OTU10) well corresponds to var. *nodulosum* (Wallr.) Lamb, described also as *S. denudatum* Flörke (Lamb, 1977), which is characterized by rare apothecia, frequent globose soralia at tips (usually present at well-developed samples) and the absence of calcium oxalate crystals in apothecia. Its podetia are simple or sparsely branched, usually in upper half. This taxon distinctly differs from Mediterranean *S. vesuvianum* s. str. in ecology: it is a boreal-montane to arctic-alpine species, occurring usually on acidic siliceous stones and rocks.

#### Additional Specimens Examined: SPAIN, Canary Islands

***El Hierro***: 1.9km NNE of Faro de Orchilla, lava field, 27°43′24.6″N, 18°8′43.4″W, alt. 280m, leg. L. Vančurová & J. Malíček 16. 5. 2013 (PRA; A315); 3km NNW of La Restinga, lava field, 27°39′57.5″N, 17°59′38.6″W, alt. 330m, leg. L. Vančurová & J. Malíček 15. 5. 2013 (PRA; A290, A291); ***El Hierro***, El Sabinar, heap of stones, soil and lava stones, 27.7454517N, 18.1229275W, alt. 665m, leg. J. Vančurová 5. 3. 2020 (PRA; A690). ***Gran Canaria***: Agaete, ancient necropolis, lava field, 28°05′49.2″N, 15°41′30.9″W, alt. 100m, leg. L. Vančurová & J. Malíček 2. 6. 2013 (PRA; A201, A202). ***La Palma***: 2.5km NE from Puerto de Naos, lava field, 28°36′17.0″N, 17°53′43.4″W, alt. 440m, leg. L. Vančurová & J. Malíček 20. 5. 2013 (PRA; A104); *Ibid.*: 28°35′55.7″N, 17°53′36.2″W, alt. 480m, leg. L. Vančurová & J. Malíček 20. 5. 2013 (PRA; A105); 3km E of El Paso, lava rock on the border of the N-facing lava field, 28°39′11.4″N, 17°51′4.3″W, alt. 860m, leg. L. Vančurová & J. Malíček 19. 5. 2013 (PRA; A97); *Ibid.*: 28°39′10.1″N, 17°51′4.3″W, alt. 865m, leg. L. Vančurová & J. Malíček 19. 5. 2013 (PRA; A98); at base of volcano San Antonio, lava stone, 28°28′39.7″N, 17°51′1.3″W, alt. 390m, leg. L. Vančurová & J. Malíček 17. 5. 2013 (PRA; A77); at base of volcano Teneguía, little lava stones around the path, 28°27′50.9″N, 17°50′43.2″W, alt. 175m, leg. L. Vančurová & J. Malíček 17. 5. 2013 (PRA; A80); Santa Cruz de La Palma, San Isidro, E-slope of mountain ridge Cumre Nueva just above the village, weathered volcanic rock, 28°37′49″N, 17°48′1″W, alt. 675m, leg. J. Vondrák 16. 3. 2014 (PRA-Vondrák 12,151; O56); Volcan Teneguía, lava stone by the path, 28°28′23″N, 17°50′50″W, alt. 340m, leg. L. Vančurová 16. 10. 2011 (PRA; L1620); volcano San Antonio, stone on the top of a volcano, 28°29′7.8″N, 17°50′59.7″W, alt. 610m, leg. L. Vančurová & J. Malíček 17. 5. 2013 (PRA; A72, A73); *Ibid.*: little lava stones around the path, 28°29′13.8″N, 17°50′56.9″W, alt. 630m, leg. L. Vančurová & J. Malíček 17. 5. 2013 (PRA; A74); *Ibid.*: 28°29′11.4″N, 17°50′59.2″W, alt. 615m, leg. L. Vančurová & J. Malíček 17. 5. 2013 (PRA; A75); *Ibid.*: 28°28′55″N, 17°50′58″W, alt. 595m, leg. L. Vančurová 16. 10. 2011 (PRA; L1616); *Ibid.*: 28°28′53″N, 17°50′44″W, alt. 480m, leg. L. Vančurová 16. 10. 2011 (PRA; L1617, L1618); volcano Teneguía, lava rock, 28°28′29.0″N, 17°51′3.7″W, alt. 380m, leg. L. Vančurová & J. Malíček 17. 5. 2013 (PRA; A78). ***Lanzarote***: Paisaje Protegido de La Geria, lava field, 28°59′1.4″N, 13°40′53.5″W, alt. 330m, leg. L. Vančurová & J. Malíček 25. 5. 2013 (PRA; A169); Parque Natural de Los Volcanes, lava field, 29°2′39.6″N, 13°42′32.4″W, alt. 240m, leg. L. Vančurová & J. Malíček 24. 5. 2013 (PRA; A162); *Ibid.*: 29°2′27.9″N, 13°43′19.6″W, alt. 255m, leg. L. Vančurová & J. Malíček 24. 5. 2013 (PRA; A163); *Ibid.*: 29°0′50.6″N, 13°43′51.5″W, alt. 300m, leg. L. Vančurová & J. Malíček 24. 5. 2013 (PRA; A164, A165); *Ibid.*: 29°2′38.5″N, 13°43′22.5″W, alt. 205m, leg. L. Vančurová & J. Malíček 24. 5. 2013 (PRA; A167). ***Tenerife***: Arguayo, near soccer field, N-facing lava field, 28°16′20.4″N, 16°48′18.8″W, alt. 960m, leg. L. Vančurová & J. Malíček 7. 5. 2013 (PRA; A254).

## Discussion

### Phycobiont Diversity in the Study Area

We found 15 species-level lineages of phycobionts in 338 lichen thalli belonging to the genera *Stereocaulon*, *Cladonia*, and *Lepraria*. Out of these 15 lineages, only eight belonged to the genus *Asterochloris*. This is a relatively surprising finding considering the results of previous studies focusing on photobiont diversity of corresponding mycobiont genera. For example, Kosecka et al. (2021) found 21 *Asterochloris* and one *Vulcanochloris* species-level lineages in Bolivia with a considerably smaller sampling of the same lichen genera. Vančurová et al. (2020) sampled only two *Stereocaulon* species-level lineages in the Swiss Alps and found 14 *Asterochloris* and three other trebouxiophycean species-level lineages. Peksa and Škaloud (2011) recovered 10 *Asterochloris* lineages associated with *Lepraria* in the Czech Republic. In the study area, globally abundant *Asterochloris* species (*Asterochloris irregularis*, *A. pseudirregularis*, *A. lobophora*, *A. phycobiontica*, *A. gaertneri*, *A. friedlii*, and others) were not detected. One of the most common *Asterochloris* species, *A. glomerata* (Pino-Bodas and Stenroos, 2020), was present in three samples and belonged to the single genotype that is known to be exclusively from the study area.

We hypothesized that the isolation of oceanic islands may explain the limited phycobiont species richness. However, the islands in Macaronesia showed higher species richness than the less isolated Sicily and Aeolian Islands. It is more likely that phycobiont distribution can be restricted by a limited number of substrate/habitat types. The main reason for the absence of many *Asterochloris* lineages might be their preference for cold and wet climates (Vančurová et al., 2018; Pino-Bodas and Stenroos, 2020). Nevertheless, there were a few cases in which phycobionts that are typical of cold climates were recorded; for example, one sample included *A. stereocaulonicola*, which was originally described in Antarctica (Kim et al., 2020). In contrast, *A. mediterranea*, the most abundant phycobiont in the study area, copes well with warm and dry climates (Moya et al., 2015; Pino-Bodas and Stenroos, 2020). Madeira, the wettest and coldest island in our study, showed the highest phycobiont species richness. Interestingly, we found all three *Vulcanochloris* species with a high haplotype diversity on the warmest and driest island in the study area, Lanzarote. This island seemed to be the hot-spot of *Vulcanochloris* diversity. In contrast, lichens associated with *Asterochloris* (including *A. mediterranea*, which performs well in warmer habitats) were extremely rare in that island. Lindgren et al. (2020) proposed a hypothesis explaining the association *between Haveochlorella* and several *Sticta* spp. on Madagascar and Reunion. A long-distance dispersal of mycobionts may have been followed by an association with locally adapted phycobionts and the subsequent diversification of mycobionts. This pattern can be true also for the relationship between *S. canariense* and *Vulcanochloris* spp.

In one case, we noticed *Myrmecia* sp. as the phycobiont of *Cladonia*. The genus *Asterochloris* has generally been considered the exclusive phycobiont of this broadly distributed mycobiont genus (Piercey-Normore and DePriest, 2001; Yahr et al., 2006; Steinová et al., 2019; Pino-Bodas and Stenroos, 2020). However, its ability to associate with *Trebouxia* and *Chloroidium* under stressful conditions has recently been demonstrated (Park et al., 2015; Osyczka et al., 2020). Moreover, Elshobary et al. (2015) discovered unknown trebouxiophycean algae as phycobionts of *Cladonia macrophylla*.

### Phycobiont Pool Sharing

Kaasalainen et al. (2021) demonstrated that cyanolichens share photobionts and revealed the presence of photobiont-mediated lichen guilds. Most of the mycobionts shared photobionts with other non-related fungal species but other photobiont-mycobiont pairs remained isolated. In our study area, the vast majority of *Cladonia* and *Lepraria* mycobionts associated with the *A. mediterranea* phycobiont. On the other hand, phycobionts of *Stereocaulon* were highly diverse. After downsampling to a sample size of 22 (the number of *Lepraria* samples) *Stereocaulon* mycobionts associated with almost two times more phycobiont species than *Cladonia* or *Lepraria* (Supplementary Figure 5). The *Stereocaulon* species-level lineages showed a high level of specificity towards their phycobionts (on the level of algal genera, but not on the level of algal species). *Stereocaulon azoreum* was associated with the same phycobionts as *Cladonia* and *Lepraria* on the Canary Islands, whereas in Madeira, it associated with another algal species. *Stereocaulon vesuvianum* and *S. canariense* were associated with *Chloroidium* spp. and *Vulcanochloris* spp., respectively. Although *Cladonia* and *Lepraria* mycobionts often grow together with *Stereocaulon* mycobionts, *S. vesuvianum* and *S. canariense* never shared phycobionts with them in our samples. Moreover, at localities where more than one thallus was sampled with *Asterochloris*, the number of species-level lineages varied. In some cases, all samples shared one species, but at other localities, each sample contained different species of *Asterochloris*.

*Stereocaulon* is not the only mycobiont genus able to associate with various phycobiont genera. The mycobiont genus *Sticta* has been shown to associate (besides cyanobacteria) with multiple trebouxiophycean algae (*Haveochlorella*, *Chloroidium*, *Symbiochloris*, and *Elliptochloris*); some *Sticta* species are specific towards particular phycobionts, but others can associate with up to three trebouxiophycean genera (Lindgren et al., 2020). Previous studies hypothesized that *Chloroidium* phycobionts are well-adapted to volcanic substrates (Vančurová et al., 2018). However, *Cladonia* mycobionts in association with *Asterochloris* can also cope with this substrate. This fact points to the role of specificity or other mycobiont features on phycobiont distribution. On the other hand, *S. canariense* was able to grow at arid localities in association with *Vulcanochloris* spp. Nevertheless, we could not find any lichen usually associated with *Asterochloris* (*Cladonia* and *Lepraria*) in the most arid parts of the study area (Fuerteventura and most of the localities on Lanzarote), even though these have previously been reported (van den Boom and Etayo, 2006). In contrast, the driest areas were dominated by lichens known to be associated with *Trebouxia* (for example, *Ramalina* spp.). The ability of some *Cladonia* species to associate with *Trebouxia* phycobionts (Osyczka et al., 2020; Shishido et al., 2021) might be helpful under such conditions (Romeike et al., 2002; de Vera, 2012; Sadowsky and Ott, 2012; Candotto-Carniel et al., 2016; Leavitt et al., 2016); however, we did not find any sample containing *Trebouxia*.

In a few cases, we found *Asterochloris* phycobionts in thalli of *S. vesuvianum* and *S. canariense*, even though these have high specificity towards other phycobiont genera. In some of these cases, we also found common phycobionts in the same thallus. In the case of *S. vesuvianum*, we repeatedly detected more than one *Chloroidium* species in a single thallus. Furthermore, we were almost unable to obtain a non-mixed sequence of phycobionts of *S. vesuvianum* at the localities on the slopes of Mt. Etna. In that area, Darienko et al. (2018) reported multiple free-living *Chloroidium* species that can coexist in *Stereocaulon* thalli. Paul et al. (2018) mentioned difficulties with Sanger sequencing as a possible indicator of algal plurality (i.e., the co-occurrence of multiple phycobionts in individual lichen thalli). Several authors found phycobionts with different ecological optima in a single thallus (Casano et al., 2011; Gasulla et al., 2020; Molins et al., 2020), which was also the case of multiple *Chloroidium* spp. in *S. vesuvianum* thalli. Vančurová et al. (2020) found additional algal phycobionts in *Stereocaulon* thalli, which were otherwise detected as the predominant phycobionts in other mycobiont species at the same locality. Therefore, we believe that phycobiont sharing is possible even in cases when the lichens differ in a predominant phycobiont. Since different lichen genera and species differ in the incidence of this phenomenon (Dal Grande et al., 2017; Smith et al., 2020), we cannot reliably estimate whether other lichen genera in the study area also show such algal plurality.

### Climatic Factors Driving Phycobiont Distribution

**Figure 10:**
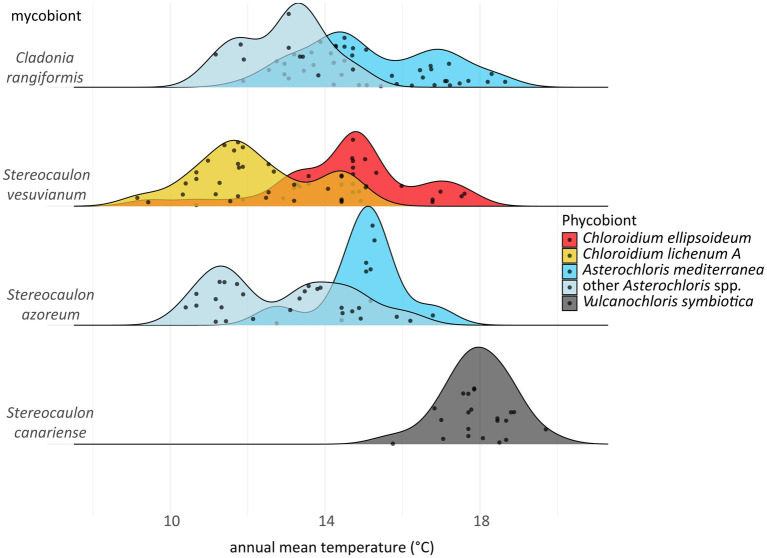
Ridgeplot depicting replacing of phycobionts along the gradient of annual mean temperature.

Even though almost all phycobionts in the study area are known to be well adapted to warm areas, they spread out along the climatic gradient. We examined the differences between those phycobionts distributed in the relatively colder and more humid areas and those taking up the widest space in the middle of the climatic niche (Figure 7B). Since both mycobionts and phycobionts contribute to the ecology of the holobiont, we tested various combinations of partners. *Asterochloris mediterranea* and other *Asterochloris* phycobionts in *Stereocaulon azoreum* differed significantly in 17 out of 19 tested climatic variables. However, this contrasting pair of phycobionts in thalli of *C. rangiformis* showed significant differences along four variables related to temperature. We considered the possibility that different thallus structure of lichen genera affected their water regime as suggested by Sadowsky et al. (2012); however, the pair of *Chloroidium* species in thalli of *S. vesuvianum* also differed somewhat in temperature-related variables. *Stereocaulon azoreum* was distributed only in Macaronesia, where temperature and precipitation are correlated. Hence, it is impossible to estimate what factor is crucial for driving the distribution of their phycobionts in this area. On the other hand, *S. vesuvianum* and *C. rangiformis* both grew on Sicily and the Aeolian Islands, where the climate characteristics are different. The three factors that were significant in all combinations were related to temperature, emphasizing the importance of this factor in the selection of the appropriate phycobiont. Several previous studies also found temperature to be the critical factor determining the distribution of phycobionts (Vančurová et al., 2018; Molins et al., 2020; Rolshausen et al., 2020). Rolshausen et al. (2020) recently proposed parallel symbiont turnover zones as demarcated regions where symbiont replacement is most likely to occur. In all gradients, this symbiont turnover zone is characterized by approximately 12°C average annual temperature. We found this pattern in all combinations, but the average annual temperature of the turnover zone was slightly higher and not uniform among the mycobiont species/genera (Figure 10). We attribute this difference to the additional influence of mycobiont selectivity. Previous studies did not determine specific turnover zones, but described the gradual change of phycobionts along the altitudinal gradient (Vargas Castillo and Beck, 2012; Gasulla et al., 2020). Moreover, Molins et al. (2020) studied *Trebouxia* phycobionts of *Buellia zoharyi* on Lanzarote, Fuerteventura, and Tenerife. They found three species that differed on their distribution ranges and the level of their tolerance to high temperatures (even under laboratory conditions). Interestingly, in corals, which is another symbiotic association similar to lichens in many aspects, the ability to cooperate with symbionts characterized by various temperature optima has been documented as an advantageous adaptive strategy (Silverstein et al., 2015). Similarly, under a scenario of global change, it may also be a critical factor for lichens (Ellis, 2019), favoring those with the ability to associate with several phycobionts covering a greater temperature gradient. However, it is possible that *Cladonia*, *Lepraria*, and *Stereocaulon* mycobionts or their phycobionts reached their maximum temperature limits, thus restricting their distribution ranges, because we did not find them on the warmest sites of the study area (Chazarra et al., 2011).

### *Stereocaulon canariense* as Endemic Species

Macaronesia is known for high level of endemism (Fernández-Palacios and Whittaker, 2008). As an oceanic island system, it is an attractive object for evolution studies (Emerson, 2002). Besides well-known examples of beetles and angiosperms, examples of lichen speciation have been documented within this area (Sérusiaux et al., 2011). *Stereocaulon canariense* was recorded only in the Canary Islands so far. However, its phycobiont, *Vulcanochloris*, has been rarely reported from other parts of the world as phycobiont of *Stereocaulon* (Kosecka et al., 2021) or other lichens (Vaiglová, 2017). The communication between Macaronesian and Mediterranean biota has been repeatedly documented (Carine et al., 2004; Vondrák et al., 2020). Consequently, other localities of this species can be found in the future. Moreover, the origin of this possibly endemic lichen can be a topic of future research.

## Data Availability Statement

The data presented in the study are deposited in GenBank, accession numbers OL622077–OL622095 and OL625120–OL625607; and Mendeley Data: http://dx.doi.org/10.17632/428v52svtp.1.

## Author Contributions

LV, JM, and PŠ designed the study. LV and JM conducted the sampling and the laboratory work. JM and JS determined the samples. LV, JS, and PŠ conducted the phylogenetic and the statistical analyses. LV and JM wrote the manuscript and produced the figures with contributions from JS and PŠ. All authors contributed to the article and approved the submitted version.

## Funding

This study was supported by Charles University Science Foundation project GAUK (grant no. 570313), Primus Research Programme of Charles University (grant no. SCI/13), and by a Long-Term Research Development grant RVO 67985939.

## Conflict of Interest

The authors declare that the research was conducted in the absence of any commercial or financial relationships that could be construed as a potential conflict of interest.

## Publisher’s Note

All claims expressed in this article are solely those of the authors and do not necessarily represent those of their affiliated organizations, or those of the publisher, the editors and the reviewers. Any product that may be evaluated in this article, or claim that may be made by its manufacturer, is not guaranteed or endorsed by the publisher.
