# Supplementary material for: Choosing the Right Life Partner: Ecological Drivers of Lichen Symbiosis
Source: Front Microbiol. 2021 Dec 14;12:769304. doi: 10.3389/fmicb.2021.769304 (PMC8712729; doi:10.3389/fmicb.2021.769304)
Supplement: Supplementary file 1 [file Data_Sheet_1.pdf]

Supplementary Material

Supplementary Table 1. GenBank accession numbers, phycobiont species-level lineage, mycobiont genus, GPS coordinates and locality of samples.

algal ITS accession	fungal ITS accession	actin accession	sample ID	phycobiont species-level lineage	mycobiont species-level lineage	GPS coordinates	
Italy, Isola di Vulcano							
**OL625283**	**OL625527**		A449	*Asterochloris mediterranea*	*Cladonia sp. 1*	38.40798	14.95492
MH415283	MH415044		A450	*Chloroidium ellipsoideum*	*S. vesuvianum*	38.39078	14.96562
MH415284	MH415045		A451	*Chloroidium ellipsoideum*	*S. vesuvianum*	38.39078	14.96562
**OL625284**	**OL625528**		A452	*Asterochloris mediterranea*	*C. foliacea 2*	38.39078	14.96562
**OL625285**	**OL625529**		A453	*Asterochloris mediterranea*	*Cladonia sp. 1*	38.39078	14.96562
**OL625286**	**OL625530**		A454	*Asterochloris mediterranea*	*L. santosii*	38.39078	14.96562
**OL625287**	**OL625531**		A456	*Asterochloris mediterranea*	*Cladonia sp. 1*	38.37617	14.98335
Italy, Isola Salina							
**OL625288**	**OL625532**		A458	*Asterochloris mediterranea*	*C. rangiformis s.l.*	38.56612	14.86415
**OL625289**	**OL625533**		A460	*Asterochloris mediterranea*	*C. foliacea 1*	38.56612	14.86415
**OL625290**	**OL625534**		A461	*Asterochloris mediterranea*	*L. santosii*	38.56612	14.86415
**OL625291**	**OL625535**		A462	*Asterochloris mediterranea*	*L. santosii*	38.56458	14.86299
**OL625292**	**OL625536**		A463	*Asterochloris mediterranea*	*C. foliacea 1*	38.56458	14.86299
**OL625293**			A464	*Asterochloris mediterranea*	*Cladonia **	38.56458	14.86299
**OL625294**	**OL625537**		A465	*Asterochloris mediterranea*	*C. rangiformis s.l.*	38.56164	14.85712
**OL625295**	**OL625538**		A466	*Asterochloris mediterranea*	*Cladonia sp. 1*	38.56164	14.85712
**OL625296**	**OL625539**		A468	*Asterochloris mediterranea*	*L. santosii*	38.56164	14.85712
**OL625297**	**OL625540**		A469	*Asterochloris mediterranea*	*C. rangiformis s.l.*	38.55874	14.85157
**OL625298**	**OL625541**		A470	*Asterochloris mediterranea*	*Cladonia sp. 1*	38.55874	14.85157
**OL625299**	**OL625542**		A471	*Asterochloris mediterranea*	*C. cervicornis*	38.55587	14.85025
**OL625300**	**OL625543**		A472	*Asterochloris mediterranea*	*C. fimbriata*	38.55587	14.85025
**OL625301**	**OL625544**		A473	*Asterochloris mediterranea*	*C. cervicornis*	38.55587	14.85025
**OL625302**	**OL625545**		A474	*Asterochloris mediterranea*	*C. rangiformis s.l.*	38.55587	14.85025

Italy, Isola Stromboli								
**OL625304**	**OL625547**		A476	*Asterochloris mediterranea*	*Cladonia sp. 1*	38.80103	15.23185
**OL625305**	**OL625548**		A477	*Asterochloris mediterranea*	*Lepraria sp. 1*	38.80103	15.23185
MH415285	MH415046		A478	*Chloroidium ellipsoideum*	*S. vesuvianum*	38.80256	15.22885
**OL625306**	**OL625549**		A480	*Asterochloris mediterranea*	*C. cenotea*	38.80208	15.22710
**OL625307**	**OL625550**		A481	*Asterochloris mediterranea*	*L. santosii*	38.80208	15.22710
**OL625308**	**OL625551**	**OL622090**	A483	*Asterochloris mediterranea*	*C. cervicornis*	38.80182	15.22599
**OL625309**	**OL625552**		A484	*Asterochloris mediterranea*	*C. rangiformis s.l.*	38.80296	15.22277
MH415286	MH415047		A485	*Chloroidium ellipsoideum*	*S. vesuvianum*	38.80081	15.22720
**OL625310**	**OL625553**		A486	*Asterochloris mediterranea*	*Cladonia sp. 1*	38.79893	15.22513
**OL625311**	**OL625554**		A487	*Asterochloris mediterranea*	*C. humilis*	38.80989	15.22325
**OL625312**	**OL625555**	**OL622091**	A488	*Asterochloris* StA10	*L. santosii*	38.80989	15.22325
Italy, Sicily							
MH415274	MH415038		A400	*Asterochloris stereocaulonicola*	*S. vesuvianum*	37.84069	15.08844
**OL625267**	**OL625512**		A405	*Asterochloris mediterranea*	*C. rangiformis s.l.*	37.77958	14.84866
**OL625268**	**OL625513**	**OL622087**	A406	*Asterochloris mediterranea*	*C. rangiformis s.l.*	37.77958	14.84866
**OL625269**	**OL625514**		A407	*Asterochloris mediterranea*	*C. humilis*	37.77958	14.84866
**OL625270**	**OL625515**		A413	*Asterochloris mediterranea*	*C. humilis*	37.70166	14.91353
**OL625271**	**OL625516**	**OL622088**	A416	*Asterochloris woessiae*	*C. foliacea 1*	37.70163	14.91368
MH415280	MH415041		A426	*Chloroidium ellipsoideum*	*S. vesuvianum*	37.65370	14.98072
**OL625272**	**OL625517**		A430	*Asterochloris mediterranea*	*C. humilis*	37.85396	15.29873
**OL625273**	**OL625518**		A431	*Asterochloris woessiae*	*C. rangiformis s.l.*	37.96275	14.61427
**OL625274**	**OL625519**		A432	*Asterochloris mediterranea*	*Cladonia sp. 1*	37.96275	14.61427
**OL625275**	**OL625520**		A433	*Asterochloris woessiae*	*C. rangiformis s.l.*	37.96275	14.61427
**OL625276**	**OL625521**		A434	*Asterochloris mediterranea*	*C. foliacea 1*	37.96303	14.61389
**OL625277**	**OL625522**		A435	*Asterochloris mediterranea*	*C. rangiformis s.l.*	37.96303	14.61389
**OL625278**	**OL625523**		A439	*Asterochloris mediterranea*	*C. humilis*	37.92881	14.67332
**OL625279**	**OL625524**		A440	*Asterochloris mediterranea*	*Cladonia sp. 1*	37.84362	14.71964
**OL625280**	**OL625525**		A441	*Asterochloris woessiae*	*C. rangiformis s.l.*	37.84362	14.71964

**OL625281**			A447	*Asterochloris mediterranea*	*Cladonia **	37.85109	14.84247
**OL625282**	**OL625526**		A448	*Asterochloris woessiae*	*C. rangiformis s.l.*	37.85109	14.84247
**OL625303**	**OL625546**	**OL622089**	A475	*Asterochloris mediterranea*	*Cladonia sp. 1*	37.97475	14.47787
**OL625313**	**OL625556**		A489	*Asterochloris mediterranea*	*C. fimbriata*	37.90840	13.97646
**OL625314**	**OL625557**		A490	*Asterochloris mediterranea*	*C. humilis*	37.90840	13.97646
**OL625315**	**OL625558**		A491	*Asterochloris mediterranea*	*C. rangiformis s.l.*	37.90840	13.97646
**OL625316**	**OL625559**	**OL622092**	A492	*Asterochloris mediterranea*	*Cladonia sp. 1*	37.89999	13.99536
**OL625317**	**OL625560**		A493	*Asterochloris mediterranea*	*C. chlorophaea 1*	37.89999	13.99536
**OL625318**	**OL625561**	**OL622093**	A494	*Asterochloris woessiae*	*C. pocillum*	37.87750	14.02926
**OL625319**			A495	*Asterochloris woessiae*	*Cladonia **	37.87750	14.02926
**OL625375**			Z11	*Asterochloris mediterranea*	*Cladonia **	37.78592	15.13031
**OL625376**			Z12	*Asterochloris mediterranea*	*Cladonia **	37.78592	15.13031
**OL625377**			Z43	*Asterochloris mediterranea*	*Cladonia **	37.78592	15.13031
**OL625378**	**OL625606**		Z7	*Chloroidium ellipsoideum*	*S. vesuvianum*	37.78592	15.13031
**OL625379**	**OL625607**		Z9	*Asterochloris mediterranea*	*Cladonia sp. 1*	37.78592	15.13031
Portugal, Madeira							
**OL625244**	**OL625490**	**OL622084**	A321	*Asterochloris* StA10	*S. azoreum*	32.75447	-17.05187
MH415230	MH414997		A322	*Asterochloris italiana*	*S. azoreum*	32.74583	-16.85887
MH415231	MH414998		A323	*Asterochloris italiana*	*S. azoreum*	32.75902	-17.05443
**OL625245**	**OL625491**		A324	*Asterochloris mediterranea*	*C. rangiformis s.l.*	32.75902	-17.05443
**OL625246**			A326	*Asterochloris mediterranea*	*Cladonia **	32.75902	-17.05443
**OL625247**	**OL625492**		A327	*Asterochloris italiana*	*C. coccifera s.l.*	32.75745	-17.05388
**OL625248**	**OL625493**		A328	*Asterochloris woessiae*	*Cladonia sp. 1*	32.75745	-17.05388
MH415232	MH414999		A329	*Asterochloris woessiae*	*S. azoreum*	32.76353	-16.92975
**OL625249**	**OL625494**		A330	*Asterochloris woessiae*	*C. coccifera s.l.*	32.76313	-16.93295
MH415234	MH415001		A331	*Asterochloris woessiae*	*Stereocaulon* OTU22	32.76313	-16.93295
**OL625250**	**OL625495**		A332	*Asterochloris italiana*	*C. cyathomorpha*	32.76313	-16.93295
MH415235	MH415002		A333	*Asterochloris woessiae*	*S. azoreum*	32.76313	-16.93295
**OL625251**	**OL625496**		A334	*Asterochloris italiana*	*C. cyathomorpha*	32.76313	-16.93295
MH415236	MH415003		A335	*Chloroidium lichenum* A	*S. vesuvianum*	32.76125	-16.93578
MH415237	MH415004		A336	*Asterochloris woessiae*	*S. azoreum*	32.76125	-16.93578
MH415238	MH415005	MH382127	A337	*Asterochloris woessiae*	*S. azoreum*	32.75315	-17.02630

**OL625252**	**OL625497**		A338	*Asterochloris mediterranea*	*C. humilis*	32.70973	-17.08567
MH415239	MH415006		A339	*Chloroidium ellipsoideum*	*S. vesuvianum*	32.72487	-16.98815
MH415240	MH415007		A340	*Chloroidium lichenum* A	*S. vesuvianum*	32.73522	-16.98548
**OL625253**	**OL625498**	**OL622085**	A342	*Asterochloris mediterranea*	*C. rangiformis s.l.*	32.75323	-17.13053
**OL625254**	**OL625499**		A343	*Asterochloris mediterranea*	*C. humilis*	32.75323	-17.13053
**OL625255**	**OL625500**		A344	*Asterochloris mediterranea*	*C. humilis*	32.76443	-17.13093
**OL625256**	**OL625501**		A346	*Asterochloris mediterranea*	*C. rangiformis s.l.*	32.70995	-16.96303
**OL625257**	**OL625502**		A347	*Asterochloris mediterranea*	*C. humilis*	32.70995	-16.96303
MH415241	MH415008		A348	*Chloroidium lichenum* A	*S. vesuvianum*	32.73937	-16.93772
MH415242	MH415009		A349	*Chloroidium lichenum* A	*S. vesuvianum*	32.74738	-16.93390
MH415244	MH415010		A350	*Asterochloris woessiae*	*S. azoreum*	32.74738	-16.93390
MH415245	MH415011		A351	*Chloroidium lichenum* A	*S. vesuvianum*	32.74932	-16.93230
MH415246	MH415012		A352	*Asterochloris italiana*	*S. azoreum*	32.74932	-16.93230
MH415247	MH415013		A353	*Chloroidium ellipsoideum*	*S. vesuvianum*	32.74932	-16.93230
MH415248	MH415014		A354	*Chloroidium lichenum* A	*S. vesuvianum*	32.75842	-16.94195
**OL625258**	**OL625503**		A355	*Asterochloris woessiae*	*C. coccifera s.l.*	32.75523	-16.93837
MH415249	MH415015		A356	*Chloroidium lichenum* B	*S. vesuvianum*	32.75523	-16.93837
MH415250	MH415016		A357	*Chloroidium ellipsoideum*	*Stereocaulon* OTU23	32.75523	-16.93837
MH415251	MH415017		A358	*Chloroidium lichenum* A	*S. vesuvianum*	32.75523	-16.93837
MH415252	MH415018	MH382128	A359	*Asterochloris woessiae*	*S. azoreum*	32.75898	-16.93945
**OL625259**	**OL625504**		A360	*Asterochloris mediterranea*	*C. rangiformis s.l.*	32.77760	-16.83943
**OL625260**	**OL625505**		A361	*Asterochloris mediterranea*	*C. humilis*	32.77760	-16.83943
**OL625261**	**OL625506**		A362	*Asterochloris mediterranea*	*C. humilis*	32.78007	-16.84313
**OL625262**	**OL625507**		A363	*Asterochloris mediterranea*	*C. humilis*	32.74583	-16.85887
**OL625263**	**OL625508**		A364	*Asterochloris mediterranea*	*Cladonia sp. 1*	32.74583	-16.85887
MH415253	MH415019		A366	*Asterochloris italiana*	*S. azoreum*	32.74153	-16.89023
**OL625264**	**OL625509**		A368	*Asterochloris mediterranea*	*C. rangiformis s.l.*	32.73235	-16.87515
**OL625265**	**OL625510**	**OL622086**	A369	*Asterochloris italiana*	*C_squamosa*	32.73235	-16.87515
MH415254	MH415020		A370	*Asterochloris italiana*	*S. azoreum*	32.72887	-16.88008
MH415255	MH415021		A371	*Asterochloris aff. italiana*	*S. azoreum*	32.72947	-16.88530

**OL625266**	**OL625511**		A374	*Asterochloris mediterranea*	*C. humilis*	32.77080	-16.94318
MH415256	MH415022		A375	*Asterochloris woessiae*	*S. azoreum*	32.77032	-16.94762
**OL625353**	**OL625586**		O135	*Asterochloris woessiae*	*C. rangiformis s.l.*	32.74011	-17.10097
**OL625354**	**OL625587**		O136	*Asterochloris woessiae*	*C. humilis*	32.74011	-17.10097
**OL625355**	**OL625588**		O137	*Asterochloris woessiae*	*Cladonia sp. 1*	32.74725	-17.07528
**OL625356**	**OL625589**		O138	*Asterochloris mediterranea*	*L. lobificans*	32.74772	-17.07492
**OL625357**	**OL625590**		O139	*Asterochloris woessiae*	*Lepraria sp. 3*	32.74772	-17.07492
**OL625358**	**OL625591**		O141	*Asterochloris mediterranea*	*Cladonia sp. 1*	32.74772	-17.07492
	**OL625592**		O143	unknown	*Lepraria sp. 2*	32.81250	-16.92750
**OL625359**	**OL625593**		O144	*Asterochloris mediterranea*	*C. rangiformis s.l.*	32.75306	-16.74278
**OL625360**			O146	*Asterochloris mediterranea*	*Cladonia **	32.81056	-17.14389
**OL625361**	**OL625594**		O147	*Asterochloris italiana*	*C_portentosa*	32.81250	-17.14389
**OL625362**	**OL625595**		O148	*Asterochloris glomerata*	*Stereocaulon* OTU3	32.81250	-17.14389
**OL625363**	**OL625596**		O149	*Asterochloris mediterranea*	*C. rangiformis s.l.*	32.81250	-17.14389
**OL625364**	**OL625597**		O150	*Asterochloris italiana*	*C. coccifera s.l.*	32.81250	-17.14389
**OL625365**	**OL625598**		O151	*Asterochloris aff. italiana*	*S. azoreum*	32.81250	-17.14389
**OL625366**	**OL625599**		O152	*Asterochloris woessiae*	*Lepraria sp. 1*	32.75917	-16.93972
**OL625367**	**OL625600**		O153	*Asterochloris woessiae*	*S. azoreum*	32.75917	-16.93972
**OL625368**	**OL625601**		O154	*Asterochloris woessiae*	*C. rangiformis s.l.*	32.75917	-16.93972
**OL625369**			O155	*Asterochloris italiana*	*Cladonia **	32.75917	-16.93972
**OL625370**	**OL625602**		O157	*Asterochloris italiana*	*S. azoreum*	32.73917	-16.93750
**OL625371**			O158	*Asterochloris italiana*	*Cladonia **	32.73917	-16.93750
**OL625372**	**OL625603**		O159	*Asterochloris mediterranea*	*L. santosii*	32.75028	-16.93889
MH415416	MH415176		O64	*Chloroidium lichenum* B	*S. vesuvianum*	32.77666	-17.08028
MH415417	MH415177		O65	*Chloroidium lichenum* A	*Stereocaulon* OTU23	32.77666	-17.08028
MH415418	MH415178		O66	*Asterochloris woessiae*	*S. azoreum*	32.76515	-16.92206
MH415419	MH415179		O67	*Asterochloris woessiae*	*S. azoreum*	32.79611	-17.18369
MH415420	MH415180		O68	*Chloroidium lichenum* A	*S. vesuvianum*	32.73904	-16.93315
MH415421	MH415181		O69	*Chloroidium lichenum* A	*S. vesuvianum*	32.76515	-16.92206
MH415422	MH415182	MH382147	O70	*Asterochloris aff. italiana*	*S. azoreum*	32.81620	-17.19001
MH415423	MH415183		O71	*Asterochloris italiana*	*S. azoreum*	32.82947	-17.19056
MH415424	MH415184		O72	*Asterochloris woessiae*	*S. azoreum*	32.84980	-17.21046
MH415425	MH415185		O73	*Asterochloris woessiae*	*Stereocaulon* OTU3	32.79611	-17.18369

MH415426	MH415186		O74	*Asterochloris woessiae*	*Stereocaulon* OTU3	32.79611	-17.18369
MH415427	MH415187	MH382148	O75	*Asterochloris glomerata*	*Stereocaulon* OTU3	32.81620	-17.19001
Spain, Canary Islands, El Hierro							
**OL625212**	**OL625463**		A279	*Asterochloris mediterranea*	*C. humilis*	27.82567	-17.93672
**OL625214**	**OL625465**		A282	*Asterochloris mediterranea*	*S. vesuvianum*	27.82453	-17.90978
**OL625215**	**OL625466**		A285	*Asterochloris mediterranea*	*S. azoreum*	27.78144	-17.95172
**OL625216**	**OL625467**		A286	*Asterochloris mediterranea*	*C. firma*	27.71611	-17.99606
**OL625217**	**OL625468**		A289	*Chloroidium ellipsoideum*	*S. vesuvianum*	27.71611	-17.99606
**OL625221**	**OL625470**		A290	*Vulcanochloris symbiotica*	*S. canariense*	27.66597	-17.99406
**OL625222**	**OL625471**		A291	*Vulcanochloris symbiotica*	*S. canariense*	27.66597	-17.99406
**OL625223**	**OL625472**		A293	*Chloroidium ellipsoideum*	*S. vesuvianum*	27.76425	-17.97028
**OL625224**	**OL625473**		A294	*Asterochloris mediterranea*	*C. foliacea 1*	27.76425	-17.97028
**OL625225**	**OL625474**		A295	*Asterochloris mediterranea*	*C. rangiformis s.l.*	27.76425	-17.97028
**OL625226**	**OL625475**		A297	*Asterochloris italiana*	*S. azoreum*	27.76256	-17.98094
**OL625227**	**OL625476**		A299	*Asterochloris mediterranea*	*C_ramulosa*	27.76053	-17.98072
**OL625231**			A300	*Asterochloris mediterranea*	*Cladonia **	27.75714	-17.98283
**OL625232**	**OL625478**		A301	*Asterochloris mediterranea*	*C. cervicornis*	27.75714	-17.98283
**OL625233**	**OL625479**		A304	*Asterochloris mediterranea*	*C. foliacea 2*	27.75556	-17.98697
**OL625234**	**OL625480**		A305	*Chloroidium ellipsoideum*	*S. vesuvianum*	27.75156	-17.99353
**OL625235**	**OL625481**		A307	*Asterochloris mediterranea*	*Cladonia sp. 1*	27.74406	-17.98633
**OL625236**	**OL625482**		A308	*Asterochloris mediterranea*	*C. rangiformis s.l.*	27.74406	-17.98633
**OL625237**	**OL625483**		A309	*Asterochloris mediterranea*	*C. umbricola 2*	27.74406	-17.98633
**OL625238**	**OL625484**		A310	*Asterochloris mediterranea*	*Cladonia sp. 2*	27.74406	-17.98633
**OL625239**	**OL625485**		A311	*Asterochloris mediterranea*	*C. rangiformis s.l.*	27.73025	-18.12044
**OL625240**	**OL625486**		A312	*Asterochloris mediterranea*	*C. humilis*	27.73025	-18.12044
**OL625241**	**OL625487**		A313	*Vulcanochloris symbiotica*	*S. canariense*	27.73025	-18.12044
**OL625242**	**OL625488**		A314	*Chloroidium ellipsoideum*	*S. vesuvianum*	27.74617	-18.12764
**OL625243**	**OL625489**		A315	*Vulcanochloris symbiotica*	*S. canariense*	27.72350	-18.14539
**OL625330**	**OL625568**		A689	*Chloroidium ellipsoideum*	*S. vesuvianum*	27.76240	-17.98091
**OL625332**	**OL625570**		A690	*Vulcanochloris symbiotica*	*S. canariense*	27.74545	-18.12293

**OL625333**	**OL625571**		A693	*Vulcanochloris canariensis*	*S. canariense*	27.71779	-18.00673
Spain, Canary Islands, Gran Canaria							
**OL625154**	**OL625411**		A171	*Asterochloris mediterranea*	*L. santosii*	27.99400	-15.53297
**OL625155**	**OL625412**		A172	*Asterochloris mediterranea*	*C. humilis*	27.99400	-15.53297
**OL625156**		**OL622081**	A173	*Asterochloris mediterranea*	*Cladonia **	27.99400	-15.53297
**OL625157**	**OL625413**		A174	*Asterochloris mediterranea*	*C. rangiformis s.l.*	27.99400	-15.53297
**OL625158**	**OL625414**		A181	*Asterochloris woessiae*	*C. rangiformis s.l.*	27.99456	-15.59456
**OL625159**	**OL625415**		A182	*Asterochloris* clade A11	*C. umbricola 1*	28.05542	-15.68911
**OL625160**	**OL625416**		A183	*Asterochloris mediterranea*	*C. rangiformis s.l.*	28.05542	-15.68911
**OL625161**	**OL625417**		A184	*Asterochloris mediterranea*	*C. rangiformis s.l.*	28.05542	-15.68911
**OL625162**	**OL625418**		A185	*Asterochloris mediterranea*	*C. humilis*	28.05542	-15.68911
**OL625163**	**OL625419**		A189	*Asterochloris mediterranea*	*C. foliacea 2*	27.91831	-15.68772
**OL625164**			A190	*Asterochloris mediterranea*	*Cladonia **	27.96825	-15.60953
**OL625165**			A192	*Asterochloris woessiae*	*Lepraria **	27.97214	-15.61378
**OL625166**			A193	*Asterochloris mediterranea*	*Cladonia **	27.92031	-15.60283
**OL625167**	**OL625420**		A194	*Myrmecia* sp.	*C. rangiformis s.l.*	27.92031	-15.60283
**OL625168**	**OL625421**		A197	*Asterochloris mediterranea*	*C. rangiformis s.l.*	28.06706	-15.66000
**OL625169**	**OL625422**		A198	*Asterochloris mediterranea*	*C. foliacea 1*	28.06706	-15.66000
**OL625170**	**OL625423**		A199	*Asterochloris mediterranea*	*Lepraria sp. 1*	28.06706	-15.66000
**OL625171**	**OL625424**		A201	*Vulcanochloris guanchorum*	*S. canariense*	28.09700	-15.69192
**OL625172**	**OL625425**		A202	*Vulcanochloris guanchorum*	*S. canariense*	28.09700	-15.69192
**OL625173**	**OL625426**		A205	*Asterochloris mediterranea*	*C. rangiformis s.l.*	28.09167	-15.59389
**OL625174**	**OL625427**		A206	*Asterochloris mediterranea*	*C. rangiformis s.l.*	28.03261	-15.45736
**OL625175**	**OL625428**	**OL622082**	A207	*Asterochloris mediterranea*	*C. foliacea 2*	28.03261	-15.45736
**OL625176**	**OL625429**		A209	*Asterochloris mediterranea*	*L. santosii*	28.03261	-15.45736
**OL625177**	**OL625430**		A210	*Asterochloris mediterranea*	*C. humilis*	28.03267	-15.45394
**OL625178**	**OL625431**		A212	*Asterochloris mediterranea*	*Cladonia sp. 1*	28.03386	-15.45811
Spain, Canary Islands, La Gomera							
**OL625122**	**OL625381**		A108	*Asterochloris italiana*	*C. umbricola 1*	28.10847	-17.24261
**OL625123**	**OL625382**		A109	*Asterochloris mediterranea*	*C. rangiformis s.l.*	28.10847	-17.24261
KP318666	**OL625383**		A110	*Asterochloris woessiae*	*Cladonia sp. 1*	28.10847	-17.24261
**OL625124**	**OL625384**		A114	*Chloroidium lichenum* B	*S. vesuvianum*	28.10647	-17.25008

KP318667	MH414986		A116	*Asterochloris woessiae*	*S. azoreum*	28.10842	-17.25361
**OL625125**		**OL622078**	A119	*Asterochloris* StA10	*Cladonia **	28.10842	-17.25361
**OL625126**	**OL625385**		A123	*Asterochloris woessiae*	*C. rangiformis s.l.*	28.10598	-17.25461
**OL625127**	**OL625386**		A124	*Asterochloris woessiae*	*Lepraria sp. 3*	28.10598	-17.25461
**OL625128**	**OL625387**		A126	*Asterochloris italiana*	*S. azoreum*	28.10953	-17.21403
**OL625129**	**OL625388**	**OL622079**	A128	*Asterochloris mediterranea*	*C. rangiformis s.l.*	28.10953	-17.21403
**OL625130**	**OL625389**		A129	*Asterochloris mediterranea*	*C. rangiformis s.l.*	28.10017	-17.18508
**OL625131**	**OL625390**		A131	*Asterochloris mediterranea*	*C. rangiformis s.l.*	28.10250	-17.18622
**OL625132**	**OL625391**		A132	*Asterochloris mediterranea*	*C. humilis*	28.10250	-17.18622
**OL625133**	**OL625392**		A134	*Chloroidium ellipsoideum*	*S. vesuvianum*	28.10982	-17.21425
**OL625134**	**OL625393**		A136	*Asterochloris mediterranea*	*L. santosii*	28.10697	-17.18794
**OL625135**	**OL625394**		A138	*Asterochloris mediterranea*	*C. humilis*	28.10694	-17.18797
**OL625136**	**OL625395**		A139	*Asterochloris mediterranea*	*Cladonia sp. 1*	28.11017	-17.19811
**OL625137**	**OL625396**		A140	*Asterochloris mediterranea*	*Cladonia sp. 2*	28.11017	-17.19811
**OL625138**	**OL625397**		A141	*Asterochloris mediterranea*	*Cladonia sp. 2*	28.11021	-17.19905
**OL625139**	**OL625398**		A143	*Asterochloris mediterranea*	*C. rangiformis s.l.*	28.16306	-17.29944
**OL625140**	**OL625399**	**OL622080**	A145	*Asterochloris mediterranea*	*C. humilis*	28.16341	-17.29943
**OL625141**	**OL625400**		A148	*Asterochloris mediterranea*	*S. azoreum*	28.12875	-17.28811
**OL625142**	**OL625401**		A149	*Asterochloris mediterranea*	*S. azoreum*	28.12875	-17.28811
**OL625143**	**OL625402**		A150	*Chloroidium ellipsoideum*	*S. vesuvianum*	28.12875	-17.28811
**OL625144**	**OL625403**		A151	*Asterochloris mediterranea*	*C. rangiformis s.l.*	28.12875	-17.28811
**OL625145**	**OL625404**		A152	*Asterochloris mediterranea*	*Lepraria sp. 1*	28.12875	-17.28811
**OL625146**			A153	*Asterochloris mediterranea*	*Cladonia **	28.13847	-17.21369
Spain, Canary Islands, La Palma							
**OL625334**	**OL625572**	**OL622094**	A70	*Asterochloris mediterranea*	*C. firma*	28.48550	-17.84992
**OL625335**	**OL625573**		A71	*Asterochloris mediterranea*	*C. rangiformis s.l.*	28.48550	-17.84992
KR952321	MH415063		A72	*Vulcanochloris symbiotica*	*S. canariense*	28.48550	-17.84992
KR952322	MH415064		A73	*Vulcanochloris symbiotica*	*S. canariense*	28.48550	-17.84992
KR952323	MH415065		A74	*Vulcanochloris symbiotica*	*S. canariense*	28.48717	-17.84914
KR952324	MH415066		A75	*Vulcanochloris symbiotica*	*S. canariense*	28.48651	-17.84979

KR952325	MH415067		A77	*Vulcanochloris symbiotica*	*S. canariense*	28.47769	-17.85036
KR952326	MH415068		A78	*Vulcanochloris symbiotica*	*S. canariense*	28.47472	-17.85103
KR952327	MH415069		A80	*Vulcanochloris symbiotica*	*S. canariense*	28.46414	-17.84533
**OL625336**	**OL625574**		A81	*Asterochloris mediterranea*	*S. azoreum*	28.80733	-17.90706
**OL625337**	**OL625575**		A83	*Asterochloris mediterranea*	*S. azoreum*	28.80733	-17.90706
**OL625338**	**OL625576**	**OL622095**	A84	*Asterochloris* StA10	*L. lobificans*	28.80733	-17.90706
**OL625339**	**OL625577**		A85	*Asterochloris mediterranea*	*C. conista*	28.80733	-17.90706
**OL625340**	**OL625578**		A87	*Asterochloris mediterranea*	*Cladonia sp. 1*	28.80925	-17.90761
**OL625341**	**OL625579**		A91	*Asterochloris mediterranea*	*S. azoreum*	28.81153	-17.91072
**OL625342**	**OL625580**		A96	*Asterochloris mediterranea*	*Cladonia sp. 1*	28.65317	-17.85119
KR952328	MH415070		A97	*Vulcanochloris symbiotica*	*S. canariense*	28.65317	-17.85119
KR952329	MH414980		A98	*Vulcanochloris canariensis*	*S. canariense*	28.65280	-17.85120
**OL625120**		**OL622077**	A101	*Asterochloris mediterranea*	*Lepraria **	28.70239	-17.85431
KR952330	MH414983		A104	*Vulcanochloris guanchorum*	*S. canariense*	28.60472	-17.89539
KR952331	MH414984		A105	*Vulcanochloris symbiotica*	*S. canariense*	28.59881	-17.89339
**OL625121**	**OL625380**		A106	*Asterochloris mediterranea*	*C. humilis*	28.59881	-17.89339
**OL625343**			KO21	*Asterochloris mediterranea*	*Cladonia **	28.71968	-17.88420
**OL625344**			KO22	*Asterochloris mediterranea*	*Cladonia **	28.71968	-17.88420
MH415312	MH415075		KO25.1	*Chloroidium lichenum* A	*S. vesuvianum*	28.76194	-17.87611
MH415313	MH415075		KO25.2	*Asterochloris mediterranea*	*S. vesuvianum*	28.76315	-17.88420
**OL625345**	**OL625581**		KO28	*Asterochloris mediterranea*	*C. rangiformis s.l.*	28.69739	-17.85299
**OL625346**	**OL625582**		KO30	*Asterochloris mediterranea*	*C. rangiformis s.l.*	28.77298	-17.81183
**OL625347**	**OL625583**		KO31A	*Asterochloris mediterranea*	*Cladonia **	28.77298	-17.81183
**OL625348**	**OL625583**		KO31B	*Asterochloris mediterranea*	*C. rangiformis s.l.*	28.77298	-17.81183
**OL625349**	**OL625583**		KO31C	*Asterochloris mediterranea*	*Cladonia **	28.77245	-17.81235
**OL625350**	**OL625584**		KO34	*Asterochloris mediterranea*	*C. rangiformis s.l.*	28.78043	-17.80287
**OL625351**			KO35	*Asterochloris mediterranea*	*Cladonia **	28.78357	-17.80065
**OL625352**	**OL625585**		KO36	*Asterochloris mediterranea*	*C. rangiformis s.l.*	28.74657	-17.76441
KR952317	MH415098		L1616	*Vulcanochloris symbiotica*	*S. canariense*	28.48194	-17.84944
KR952318	MH415099		L1617	*Vulcanochloris symbiotica*	*S. canariense*	28.48139	-17.84556
KR952319	MH415100		L1618	*Vulcanochloris symbiotica*	*S. canariense*	28.48139	-17.84556
KR952320	MH415101		L1620	*Vulcanochloris canariensis*	*S. canariense*	28.47306	-17.84722

MH415341	MH415102	L1621	*Chloroidium lichenum* A	*S. vesuvianum*	28.60889	-17.83556
MH415342	MH415103	L1623	*Chloroidium lichenum* A	*S. vesuvianum*	28.60806	-17.83833
MH415343	MH415104	L1624	*Chloroidium lichenum* A	*S. vesuvianum*	28.60806	-17.83833
MH415344	MH415105	L1625	*Chloroidium lichenum* A	*S. vesuvianum*	28.60583	-17.84028
MH415345	MH415106	L1626	*Chloroidium lichenum* A	*S. vesuvianum*	28.59750	-17.84056
MH415346	MH415107	L1627	*Chloroidium lichenum* A	*S. vesuvianum*	28.59750	-17.84056
MH415347	MH415108	L1628	*Chloroidium lichenum* A	*S. vesuvianum*	28.58333	-17.83333
MH415348	MH415109	L1630	*Chloroidium lichenum* A	*S. vesuvianum*	28.56667	-17.83333
MH415349	MH415110	L1632	*Chloroidium lichenum* A	*S. vesuvianum*	28.56667	-17.83333
MH415350	MH415111	L1633	*Chloroidium lichenum* A	*S. vesuvianum*	28.56667	-17.83333
MH415351	MH415112	L1634	*Chloroidium lichenum* A	*S. vesuvianum*	28.57333	-17.84056
MH415352	MH415113	L1636	*Chloroidium lichenum* A	*S. vesuvianum*	28.51861	-17.83472
MH415353	MH415114	L1637	*Chloroidium lichenum* A	*S. vesuvianum*	28.75889	-17.88111
MH415354	MH415115	L1638	*Chloroidium ellipsoideum*	*S. vesuvianum*	28.76083	-17.87278
MH415355	MH415116	L1639	*Chloroidium ellipsoideum*	*S. vesuvianum*	28.64861	-17.83833
**OL625373**	**OL625604**	O56	*Vulcanochloris symbiotica*	*S. canariense*	28.63028	-17.80028
**OL625374**	**OL625605**	O63	*Asterochloris mediterranea*	*S. azoreum*	28.81667	-17.81194
Spain, Canary Islands, Lanzarote						
**OL625147**	**OL625405**	A159	*Asterochloris mediterranea*	*C. rangiformis s.l.*	29.13131	-13.51464
**OL625148**		A162	*Vulcanochloris symbiotica*	*S. canariense*	29.04433	-13.70900
**OL625149**	**OL625406**	A163	*Vulcanochloris canariensis*	*S. canariense*	29.04108	-13.72211
**OL625150**	**OL625407**	A164	*Vulcanochloris guanchorum*	*S. canariense*	29.01406	-13.73097
**OL625151**	**OL625408**	A165	*Vulcanochloris canariensis*	*S. canariense*	29.01406	-13.73097
**OL625152**	**OL625409**	A167	*Vulcanochloris symbiotica*	*S. canariense*	29.04403	-13.72292
**OL625153**	**OL625410**	A169	*Vulcanochloris symbiotica*	*S. canariense*	28.98372	-13.68153
Spain, Canary Islands, Tenerife						
**OL625198**	**OL625449**	A25	*Asterochloris woessiae*	*C. rangiformis s.l.*	28.33243	-16.76208
**OL625204**	**OL625455**	A26	*Asterochloris mediterranea*	*C. rangiformis s.l.*	28.33243	-16.76208
**OL625210**	**OL625461**	A27	*Asterochloris mediterranea*	*C. humilis*	28.33243	-16.76208
**OL625213**	**OL625464**	A28	*Asterochloris mediterranea*	*C. humilis*	28.33243	-16.76208

**OL625218**	**OL625469**	A29_1	*Chloroidium ellipsoideum*	*S. vesuvianum*	28.33243	-16.76208
**OL625219**	**OL625469**	A29_2	*Chloroidium ellipsoideum*	*S. vesuvianum*	28.33243	-16.76208
**OL625220**	**OL625469**	A29_4	*Chloroidium ellipsoideum*	*S. vesuvianum*	28.33243	-16.76208
**OL625228**	**OL625477**	A30_3	*Chloroidium ellipsoideum*	*S. vesuvianum*	28.33243	-16.76208
**OL625229**	**OL625477**	A30_4	*Chloroidium ellipsoideum*	*S. vesuvianum*	28.33243	-16.76208
**OL625230**	**OL625477**	A30_6	*Chloroidium ellipsoideum*	*S. vesuvianum*	28.33243	-16.76208
**OL625320**	**OL625562**	A61	*Asterochloris mediterranea*	*C. humilis*	28.35774	-16.51360
**OL625321**	**OL625563**	A62	*Asterochloris mediterranea*	*S. azoreum*	28.35774	-16.51360
**OL625322**	**OL625564**	A63	*Asterochloris mediterranea*	*C. rangiformis s.l.*	28.35774	-16.51360
**OL625323**	**OL625565**	A64	*Asterochloris mediterranea*	*C. humilis*	28.35774	-16.51360
**OL625324**	**OL625566**	A65_1	*Chloroidium lichenum* A	*S. vesuvianum*	28.35774	-16.51360
**OL625325**	**OL625566**	A65_2	*Chloroidium lichenum* A	*S. vesuvianum*	28.35774	-16.51360
KP318665		A66	*Asterochloris woessiae*	*Cladonia **	28.35774	-16.51360
**OL625326**	**OL625567**	A67_3	*Chloroidium lichenum* A	*S. vesuvianum*	28.35774	-16.51360
**OL625327**	**OL625567**	A67_4	*Chloroidium ellipsoideum*	*S. vesuvianum*	28.35774	-16.51360
**OL625328**	**OL625567**	A67_5	*Chloroidium lichenum* A	*S. vesuvianum*	28.35774	-16.51360
**OL625329**	**OL625567**	A67_6	*Chloroidium lichenum* A	*S. vesuvianum*	28.35774	-16.51360
**OL625331**	**OL625569**	A69	*Asterochloris mediterranea*	*C. pyxidata*	28.35774	-16.51360
**OL625179**	**OL625432**	A213	*Asterochloris mediterranea*	*L. santosii*	28.17157	-16.63677
**OL625180**	**OL625433**	A214	*Chloroidium ellipsoideum*	*S. vesuvianum*	28.29032	-16.76579
**OL625181**	**OL625434**	A215	*Chloroidium lichenum* A	*S. vesuvianum*	28.29032	-16.76579
**OL625182**	**OL625435**	A217	*Chloroidium lichenum* A	*S. vesuvianum*	28.29032	-16.76579
**OL625183**	**OL625436**	A219	*Chloroidium lichenum* A	*S. vesuvianum*	28.30321	-16.79238
**OL625184**	**OL625437**	A222	*Chloroidium ellipsoideum*	*S. vesuvianum*	28.30029	-16.76750
**OL625185**	**OL625438**	A225	*Chloroidium ellipsoideum*	*S. vesuvianum*	28.31480	-16.74970
**OL625186**	**OL625439**	A226	*Chloroidium ellipsoideum*	*S. vesuvianum*	28.31480	-16.74970
**OL625187**	**OL625440**	A227	*Asterochloris mediterranea*	*C. humilis*	28.31480	-16.74970
**OL625188**	**OL625441**	A228	*Asterochloris mediterranea*	*C. humilis*	28.31480	-16.74970
**OL625189**	**OL625442**	A229	*Asterochloris mediterranea*	*C. humilis*	28.31480	-16.74970
**OL625190**	**OL625443**	A232	*Asterochloris mediterranea*	*C. humilis*	28.32101	-16.73141
**OL625191**		A234	*Asterochloris mediterranea*	*Lepraria **	28.32101	-16.73141
**OL625192**	**OL625444**	A237	*Asterochloris mediterranea*	*C. humilis*	28.33833	-16.83467

**OL625193**	**OL625445**		A239	*Asterochloris mediterranea*	*C. humilis*	28.35774	-16.51360
**OL625194**	**OL625446**		A240	*Asterochloris mediterranea*	*C. humilis*	28.34771	-16.50358
**OL625195**	**OL625447**	**OL622083**	A241	*Asterochloris mediterranea*	*C. rangiformis s.l.*	28.33584	-16.82626
**OL625196**	**OL625448**		A242	*Asterochloris mediterranea*	*C. cervicornis*	28.33584	-16.82626
**OL625197**			A244	*Asterochloris mediterranea*	*Cladonia **	28.35998	-16.76821
**OL625199**	**OL625450**		A251	*Asterochloris mediterranea*	*S. azoreum*	28.41114	-16.41708
**OL625200**	**OL625451**		A252	*Asterochloris mediterranea*	*C. rangiformis s.l.*	28.41114	-16.41708
**OL625201**	**OL625452**		A253	*Asterochloris mediterranea*	*C. fimbriata*	28.41114	-16.41708
**OL625202**	**OL625453**		A254	*Asterochloris mediterranea*	*S. canariense*	28.27233	-16.80522
**OL625203**	**OL625454**		A259	*Asterochloris glomerata*	*C. chlorophaea 2*	28.53244	-16.28042
**OL625205**	**OL625456**		A260	*Asterochloris mediterranea*	*C. humilis*	28.53244	-16.28042
**OL625206**	**OL625457**		A264	*Asterochloris mediterranea*	*Cladonia sp. 1*	28.53047	-16.28003
**OL625207**	**OL625458**		A266	*Asterochloris mediterranea*	*C. rangiformis s.l.*	28.55911	-16.17108
**OL625208**	**OL625459**		A267	*Asterochloris mediterranea*	*Cladonia sp. 1*	28.55911	-16.17108
**OL625209**	**OL625460**		A268	*Asterochloris mediterranea*	*C. humilis*	28.55911	-16.17108
**OL625211**	**OL625462**		A274	*Asterochloris mediterranea*	*C. firma*	28.56519	-16.29792

* Mycobiont ITS sequence not available.

Supplementary Table 2. Total number of sequences included in particular alignments, newly obtained sequences, previously published sequences originated from the study area, reference sequences retrieved from GenBank, and unique sequences after deleting identical ones.

Alignment	Sequences total	Newly obtained	Previously published (from the study area)	Reference sequences	Unique sequences
Asterochloris ITS	316	216	27 (Škaloud et al., 2015; Vančurová et al., 2015, 2018)	73	98
Asterochloris actin	89	19	4 (Vančurová et al., 2018)	66	89
Chloroidium ITS	81	28	36 (Vančurová et al., 2018)	17	30
Vulcanochloris ITS	34	15	15 (Vančurová et al., 2015)	4	20
Stereocaulon ITS	152	52	75 (Vančurová et al., 2018)	25	75
Cladonia ITS	194	156	0	38	75
Lepraria ITS	50	20	0	30	40

Supplementary Table 3. Substitution models selected using the Bayesian information criterion (BIC) as implemented in JModelTest2 (Guindon and Gascuel, 2003; Darriba et al., 2012).

partition	*Stereocaulon*	*Cladonia*	*Lepraria*	*Asterochloris*	*Chloroidium*	*Vulcanochloris*
ITS1 rDNA	T92 + Γ (α=0.46)	K2 + I	K2 + Γ (α=1.15)	K2 + Γ (α=0.61)	T92 + Γ (α=0.6)	T92
5.8 S rDNA	K2 + Γ (α=0.05)	JC	JC + Γ (α=0.05)	JC	JC	JC
ITS2 rDNA	T92 + Γ (α=0.59)	K2 + Γ (α=0.69)	K2 + Γ (α=0.55)	K2 + Γ (α=0.31)	T92 + Γ (α=0.65)	JC + Γ (α=0.05)
intron 206 of actin type I gene				JC		
exon part of actin type I gene				K2 + Γ (α=0.23)		
intron 248 of actin type I gene				K2		

Supplementary Table 4. *Asterochloris* reference sequences retrieved from GenBank with accession numbers.

ITS accession	actin accession	sample ID	species-level lineage
AM905998	AM906026	Peksa 498	*Asterochloris glomerata*
AF345382	AM906024	UTEX 895	*Asterochloris glomerata*
MH415370	MH382143	VancurovaL992	*Asterochloris irregularis*
AF345411	AM906027	UTEX 2236	*Asterochloris irregularis*
MH415367	MH382142	VancurovaL988	*Asterochloris pseudoirregularis*
DQ229885	DQ229888	Talbot 281	*Asterochloris pseudoirregularis*
AM906012	AM906041	UTEX902	*Asterochloris magna*
AF345440	AM906018	UTEX911	*Asterochloris erici*
MH415428	MH382149	VancurovaO76	StA1
HE803036	MH382118	IH23	I2
MH415296	MH382135	VancurovaA504	
HE803038	KP318682	IH20	clade 9
MW043523	MW073611	CL105	OTU14
MW847827		Kukwa 16553	Bol 5
MW847847		UGDA L 18555	Bol 4
MW847820		Kukwa 15135	Bol 3
MW043562	MW073638	CL176	OTU25
MW043566	MW073641	CL188	OTU25
DQ229884	DQ229896	Nelsen 2181b	S1
MW043643	MW073700	CL320	OTU24
EU008684	EU008711	L54	
FM945380	FM955675	Peksa 787	clade 8
FM945358	FM955674	Peksa 796	clade 8
MW043500	MW073601	3CONIST	OTU21
DQ229877	DQ229898	Nelsen 3974	*Asterochloris friedlii*
AM905995	AM906021	Peksa 235	*Asterochloris friedlii*
HE803033	MH382119	IH31	I1
HE803029	MH382117	I6	I1
MH415257	MH382129	VancurovaA378	StA2
MH415269	MH382131	VancurovaA392	StA2
MW847845	MW845968	Kukwa19499	Bol 6
DQ229886	DQ229897	Talbot KIS 187	S3
AM900492	AM906045	Bayerová 3401	*Asterochloris woessiae*
MH415438	MH382150	VancurovaO98	StA3
MW847825	MW845961	Kukwa16204	Bol 1
MW847831	MW845963	Kukwa16828	Bol 1
KX051236	KX051240	KGS064B	*Asterochloris sejongensis*
KX051235	KX051239	KGS007A	*Asterochloris sejongensis*
MW847836		Kukwa16907a	Bol 9
MT066266	MT093216	VancurovaA526	StA9
KP257384	KP257351	C19	*Asterochloris mediterranea*
EU008690	EU008715	L60	URa14

MH415220	MH382124	VancurovaA14	
MW043556	MW073633	CL166	OTU14
AM906001	AM906030	CCAP 219/5B	*Asterochloris italiana*
MW043630	MW073687	CL296	*Asterochloris italiana*
DQ229882	DQ229893	Talbot 400	StA4
MH415329	MH382137	VancurovaL1074	StA4
AM900490	AM906042	SAG 26.81	*Asterochloris phycobiontica*
MH415414	MH382146	VancurovaO50	StA5
MH415366	MH382141	VancurovaL958	StA5
FN556044	KP318679	Peksa 866	*Asterochloris lobophora*
AM906008	AM906037	Peksa 166	*Asterochloris lobophora*
FM945378	FM955677	Peksa 921	clade 12
DQ229887		Talbot101	clade 12
MW847810	MW845958	Kukwa14827	Bol 7
MW847823		Kukwa15988	Bol 2
AM905992	AM906017	Peksa 186	*Asterochloris echinata*
FM955667	FM955671	Peksa 551	*Asterochloris echinata*
KP318676	KP318681	Peksa 495	A4
AM905993	AM906019	UTEX 1714	*Asterochloris excentrica*
MW847848		UGDA L 18556	Bol 8
AM906002	AM906031	Peksa 183	*Asterochloris leprarii*
MH415226	MH382125	VancurovaA3	*Asterochloris stereocaulonicola*
FN556035	FN556048	Peksa 860	*Asterochloris stereocaulonicola*
MH415216	MH382120	VancurovaA1	StA6
MH415219	MH382123	VancurovaA13	StA7
MH415229	MH382126	VancurovaA319	StA8
MH415288	MH382133	VancurovaA498	StA8
FM955669	FM955673	Peksa 900	*Asterochloris gaertneri*
AM905997	AM906023	Peksa 236	*Asterochloris gaertneri*
FN556042	FN556051	Peksa 873	A11
FN556043	FN556052	Peksa 870	A11

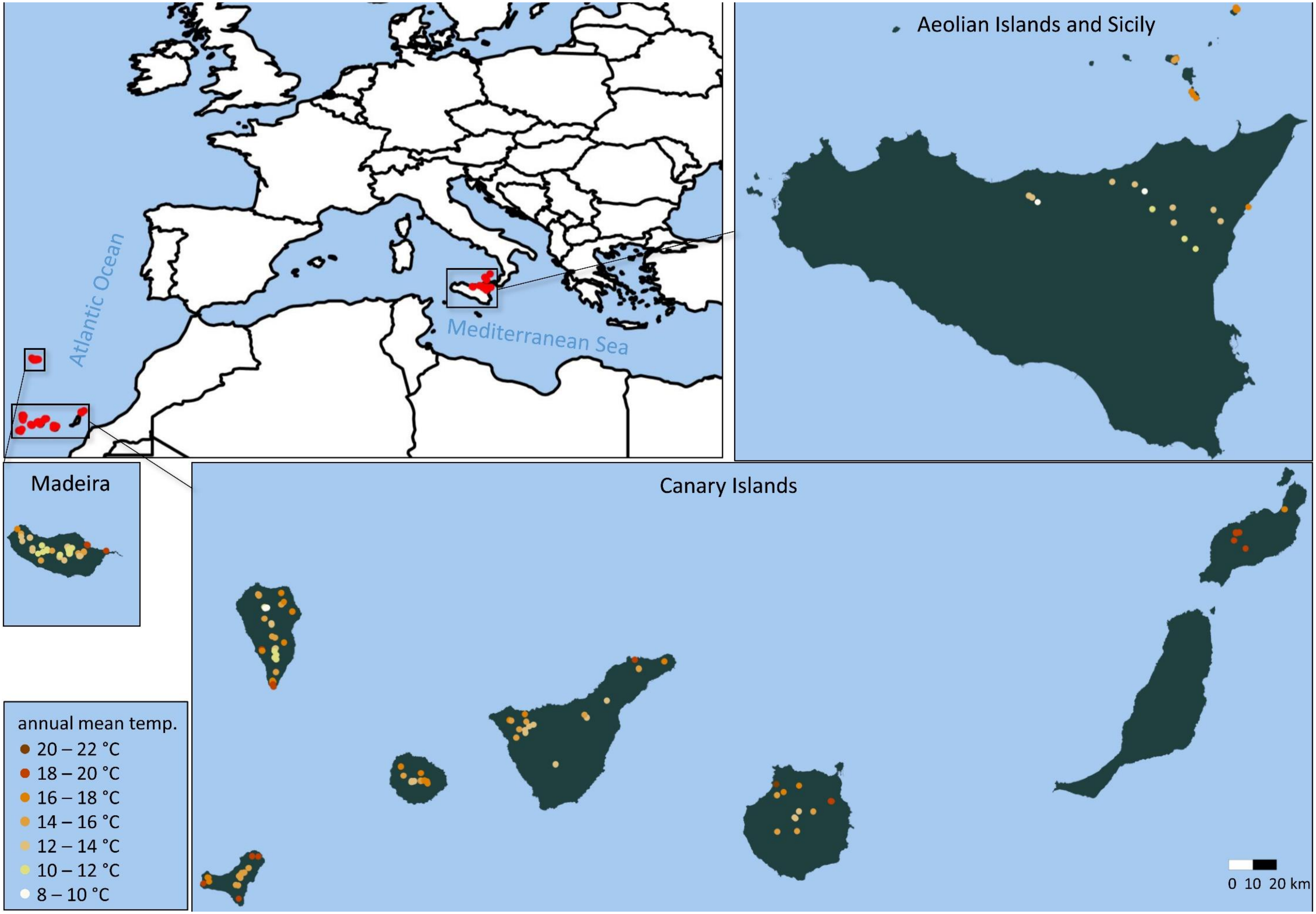
Aeolian Islands and Sicily
Atlantic Ocean
Mediterranean Sea
Madeira
Canary Islands
annual mean temp.
20 – 22 °C
18 – 20 °C
16 – 18 °C
14 – 16 °C
12 – 14 °C
10 – 12 °C
8 – 10 °C
0 10 20 km

Supplementary Figure 1. Location of the sampling sites. The annual mean temperature at the locality is indicated by the color of the dot.

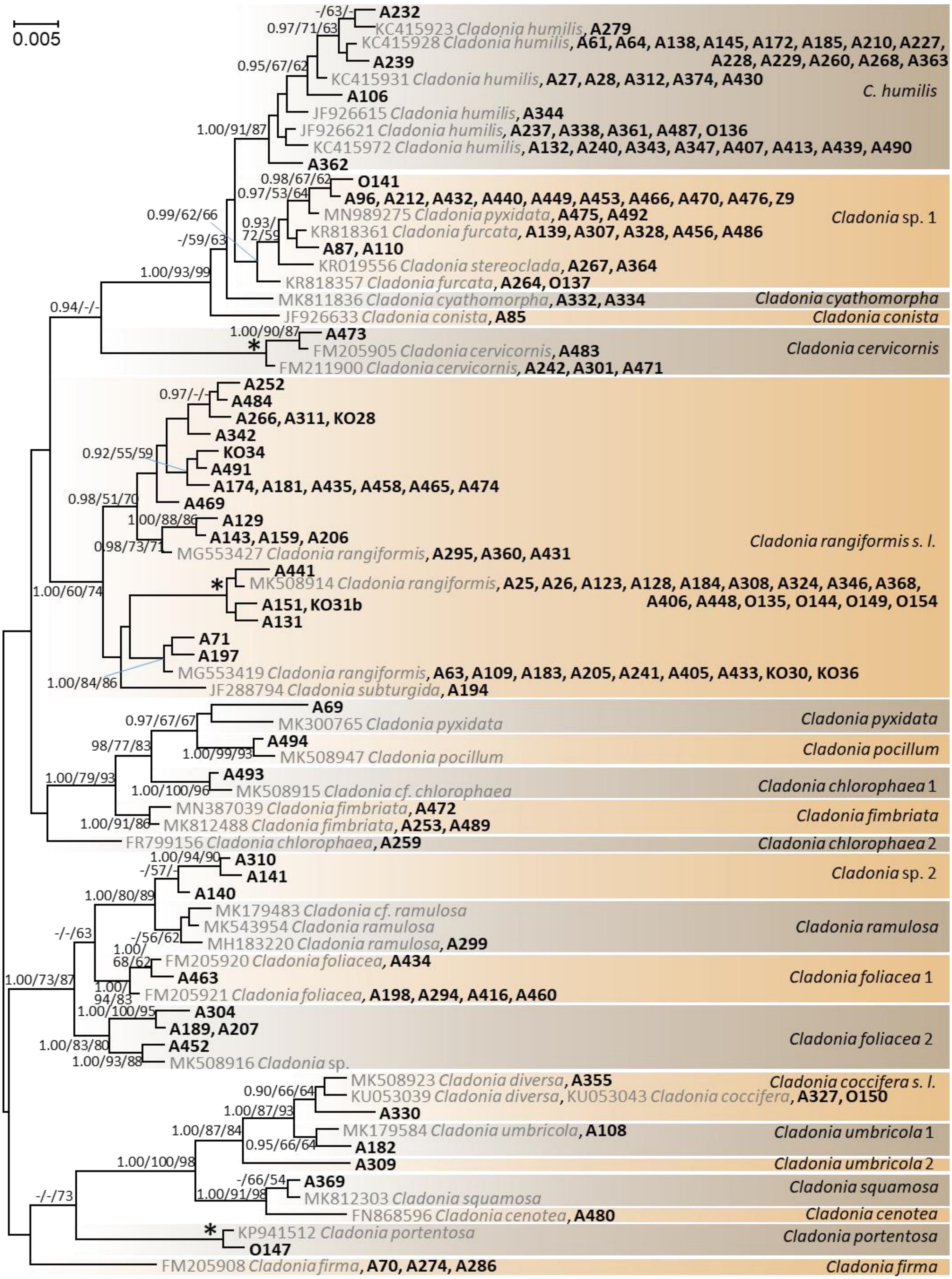
0.005
-/63/-
A232
0.97/71/63
KC415923 Cladonia humilis, A279
KC415928 Cladonia humilis, A61, A64, A138, A145, A172, A185, A210, A227, A228, A229, A260, A268, A363
A239
0.95/67/62
KC415931 Cladonia humilis, A27, A28, A312, A374, A430
A106
C. humilis
JF926615 Cladonia humilis, A344
1.00/91/87
JF926621 Cladonia humilis, A237, A338, A361, A487, O136
KC415972 Cladonia humilis, A132, A240, A343, A347, A407, A413, A439, A490
A362
0.98/67/62
O141
0.97/53/64
A96, A212, A432, A440, A449, A453, A466, A470, A476, Z9
0.99/62/66
MN989275 Cladonia pyxidata, A475, A492
Cladonia sp. 1
0.93/ 72/59
KR818361 Cladonia furcata, A139, A307, A328, A456, A486
-/59/63
A87, A110
KR019556 Cladonia stereoclada, A267, A364
1.00/93/99
KR818357 Cladonia furcata, A264, O137
MK811836 Cladonia cyathomorpha, A332, A334
Cladonia cyathomorpha
0.94/-/-
JF926633 Cladonia conista, A85
Cladonia conista
1.00/90/87
A473
*
FM205905 Cladonia cervicornis, A483
Cladonia cervicornis
FM211900 Cladonia cervicornis, A242, A301, A471
A252
0.97/-/-
A484
A266, A311, KO28
A342
0.92/55/59
KO34
A491
A174, A181, A435, A458, A465, A474
0.98/51/70
A469
1.00/88/86
A129
A143, A159, A206
Cladonia rangiformis s. l.
0.98/73/71
MG553427 Cladonia rangiformis, A295, A360, A431
A441
*
MK508914 Cladonia rangiformis, A25, A26, A123, A128, A184, A308, A324, A346, A368, A406, A448, O135, O144, O149, O154
1.00/60/74
A151, KO31b
A131
A71
A197
MG553419 Cladonia rangiformis, A63, A109, A183, A205, A241, A405, A433, KO30, KO36
1.00/84/86
JF288794 Cladonia subturgida, A194
A69
0.97/67/67
MK300765 Cladonia pyxidata
Cladonia pyxidata
98/77/83
A494
1.00/99/93
MK508947 Cladonia pocillum
Cladonia pocillum
1.00/79/93
A493
1.00/100/96
MK508915 Cladonia cf. chlorophaea
Cladonia chlorophaea 1
MN387039 Cladonia fimbriata, A472
1.00/91/86
MK812488 Cladonia fimbriata, A253, A489
Cladonia fimbriata
FR799156 Cladonia chlorophaea, A259
Cladonia chlorophaea 2
1.00/94/90
A310
-/57/-
A141
Cladonia sp. 2
1.00/80/89
A140
MK179483 Cladonia cf. ramulosa
-/-/63
MK543954 Cladonia ramulosa
Cladonia ramulosa
-/56/62
MH183220 Cladonia ramulosa, A299
1.00/ 68/62
FM205920 Cladonia foliacea, A434
1.00/73/87
A463
Cladonia foliacea 1
1.00/ 94/83
FM205921 Cladonia foliacea, A198, A294, A416, A460
1.00/100/95
A304
A189, A207
Cladonia foliacea 2
1.00/83/80
A452
1.00/93/88
MK508916 Cladonia sp.
MK508923 Cladonia diversa, A355
Cladonia coccifera s. l.
0.90/66/64
KU053039 Cladonia diversa, KU053043 Cladonia coccifera, A327, O150
1.00/87/93
A330
1.00/87/84
MK179584 Cladonia umbricola, A108
Cladonia umbricola 1
0.95/66/64
A182
1.00/100/98
A309
Cladonia umbricola 2
-/66/54
A369
Cladonia squamosa
1.00/91/98
MK812303 Cladonia squamosa
-/-/73
FN868596 Cladonia cenotea, A480
Cladonia cenotea
*
KP941512 Cladonia portentosa
Cladonia portentosa
O147
FM205908 Cladonia firma, A70, A274, A286
Cladonia firma

Supplementary Figure 2. Phylogenetic hypothesis (unrooted tree) of *Cladonia* resulting from Bayesian analysis of ITS rDNA. Values at the nodes indicate the statistical supports of Bayesian posterior probability (left), maximum-likelihood bootstrap (middle) and maximum parsimony bootstrap (right). Fully supported branches (1.0/100/100) are marked with an asterisk. Scale bar shows the estimated number of substitutions per site. Newly obtained sequences are marked in bold.

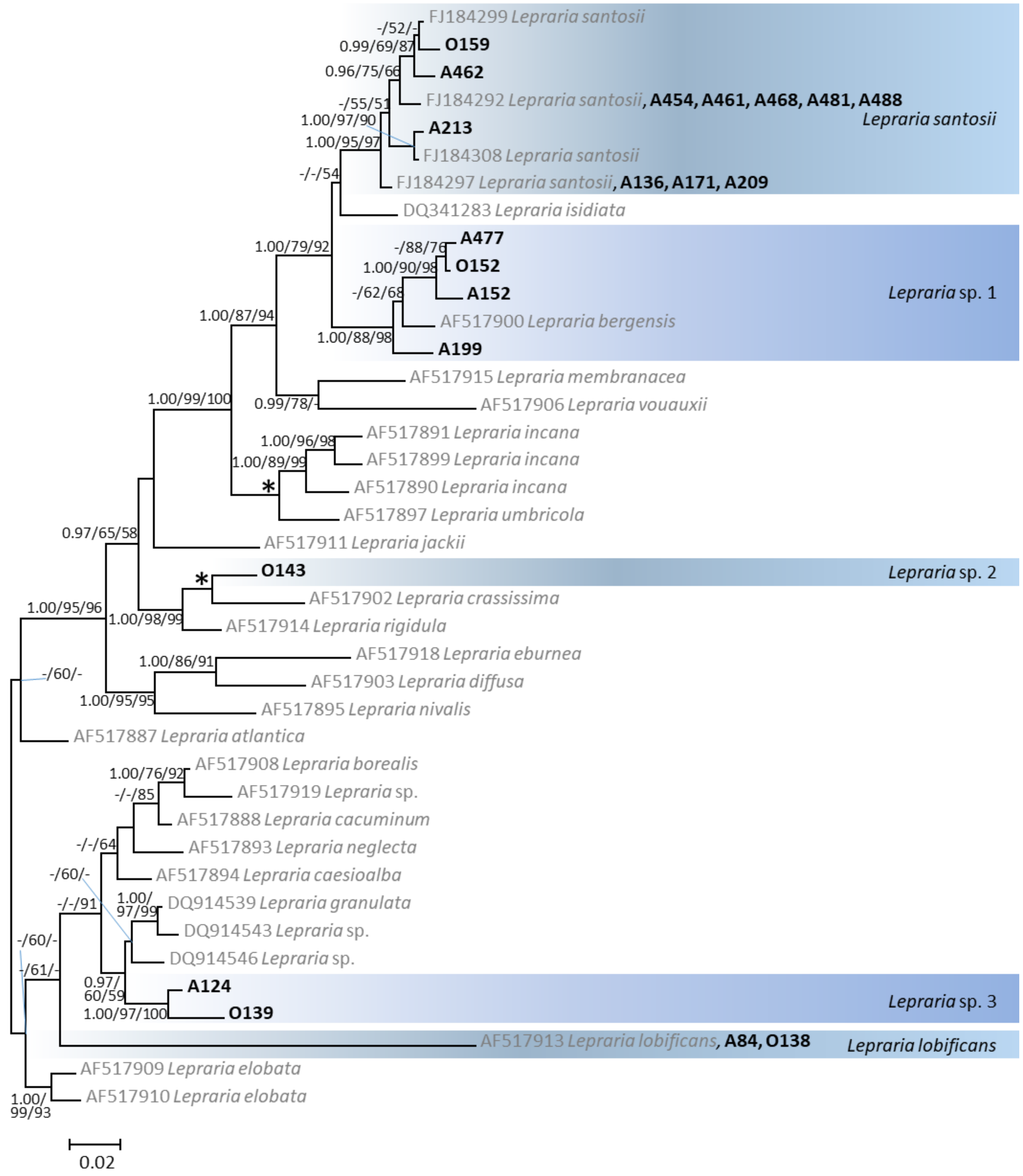

Supplementary Figure 3. Phylogenetic hypothesis (unrooted tree) of *Lepraria* resulting from Bayesian analysis of ITS rDNA. Values at the nodes indicate the statistical supports of Bayesian posterior probability (left), maximum-likelihood bootstrap (middle) and maximum parsimony bootstrap (right). Fully supported branches

(1.0/100/100) are marked with an asterisk. Scale bar shows the estimated number of substitutions per site. Newly obtained sequences are marked in bold.

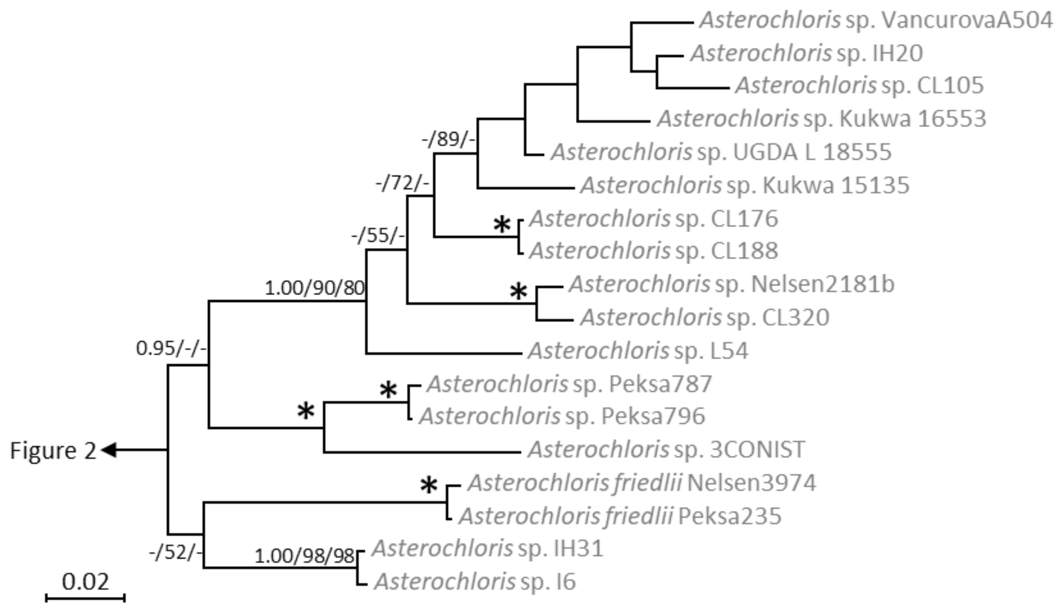

Supplementary Figure 4. Part of phylogenetic tree of *Asterochloris* (Figure 2) containing solely reference sequences (accession numbers are listed in Supplementary Table 4). Values at the nodes indicate the statistical supports of Bayesian posterior probability (left), maximum-likelihood bootstrap (middle) and maximum parsimony bootstrap (right). Fully supported branches (1.0/100/100) are marked with an asterisk. Scale bar shows the estimated number of substitutions per site.

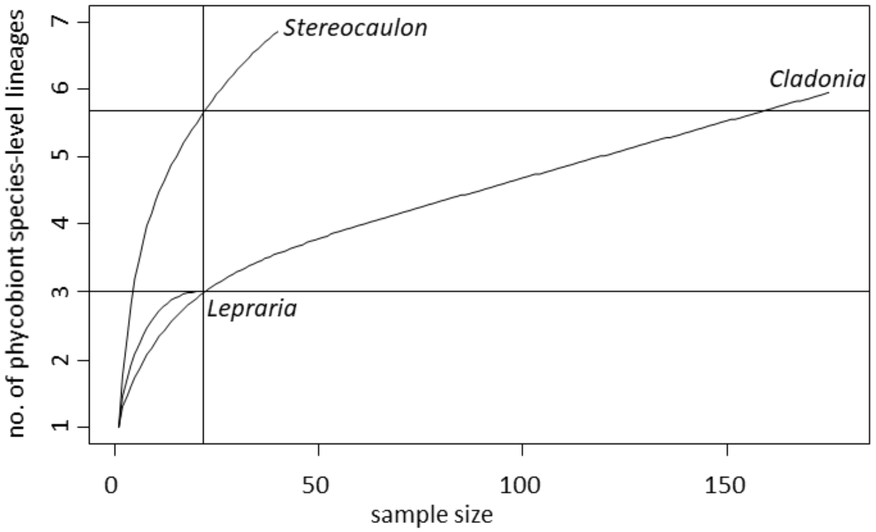

Supplementary Figure 5. Rarefaction curves for three mycobiont genera. Vertical line is drawn at smallest sample size in the data set with horizontal lines for the rarefied number of species-level lineages of associated phycobionts.
